# Sustainable supply chain practices of local versus international hotels: multiple case studies in Egypt

**DOI:** 10.1038/s41598-025-18819-9

**Published:** 2025-09-15

**Authors:** Mahmoud Ahmed Abdo Hamza, Nazaré Rego

**Affiliations:** 1 College of International Transport and Logistics, Arab Academy for Science, Technology and Maritime Transport, Sadat Road, P.O. Box 11, Aswan, Egypt; 2 Centre for Research in Economics and Management (NIPE), School of Economics and Management, University of Minho, Gualtar Campus, 4710-057 Braga, Portugal

**Keywords:** Sustainable supply chain practices, Sustainable hospitality supply chain management, Hotels, Stakeholder theory, Sustainability, Environmental impact

## Abstract

This study examines the adoption of sustainable supply chain practices (SSCPs) among three hotel categories: international chain hotels, 5-star local chains, and 4-star local chains located in Egypt. It aims to identify the drivers, barriers, and variations in the implementation of these practices. A qualitative multi-case study methodology was employed, involving in-depth interviews with managers at nine hotels. The study also presents a theoretical framework developed from stakeholder theory that illustrates how stakeholder pressures and organizational capacities drive or hinder the adoption of SSCPs and how these practices contribute to broader sustainability objectives. Significant variations were observed in SSCPs. International chains implement advanced practices such as comprehensive waste management and energy-efficiency programs. 5-star local chains focus on cost-saving initiatives and basic recycling, while 4-star local chains primarily adopt simple waste reduction and local sourcing. Differences in barriers also exist: 4-star local chains face more financial and knowledge limitations, while international chains face additional challenges related to supplier engagement and infrastructure. Hotel owners and managers can enhance sustainability by providing staff training, fostering collaboration with suppliers, and strategically allocating resources. Such activities are crucial for local chains with limited resources. This research fills gaps in the literature by exploring SSCPs in a developing-country context and integrating stakeholder theory to uncover both normative and instrumental drivers of SSCPs. It presents a conceptual framework that links SSCPs with drivers, barriers, and outcomes, providing a practical guide for hotels to enhance their sustainability.

**Supplementary Information:**

The online version contains supplementary material available at 10.1038/s41598-025-18819-9.

## Introduction

Sustainable supply chain management (SSCM) has become a central focus in organizational strategy, as companies increasingly recognize the need to integrate ecological and social considerations into their operations^1,2^. While some scholars suggest that sustainability may even surpass profitability in importance^3^, embedding sustainable innovations within supply networks remains a relatively new and evolving practice that challenges organizations to rethink their established systems^1^.

Academic research has made significant progress in identifying the core components of sustainable supply chains, as well as the triple-bottom-line model, which encompasses environmental, social, and economic outcomes^4,5^. Nonetheless, most empirical research has focused on the manufacturing sector, while service-related sectors, such as hospitality, have received little attention^6–8^. Although the impact of SSCM on sustainability performance has been associated with positive findings in studies on hotels and healthcare settings, most studies in these contexts are scarce in terms of well-grounded theory and depth^9–12^. The hospitality and tourism industries are under increasing scrutiny from stakeholders due to their environmental footprints and social responsibilities^13,14^. Addressing sustainability challenges in these sectors is vital not only for environmental stewardship but also for social equity and long-term profitability. Creative solutions, such as ecotourism, underscore the interconnectedness of hospitality and tourism, as well as the potential for shared sustainability benefits^15^.

Hotels are one of the most resource-intensive institutions in the Middle East and North Africa (MENA) region, with water and energy consumption at perilously high levels, CO₂ emissions into the atmosphere, and sustainability initiatives that both harm the environment and undermine the eco-summit. However, some hotels focus on cutting costs, while others in the West emphasize that it is shareholders’ responsibility to promote eco-friendly practices^16^. In the context of (I) the economic importance of the Egyptian hotel industry and (II) the environmental sensitivity of hotel accommodations, it is inevitable that Egypt’s hotel sector is built on its unique heritage and natural resources. The government has responded to the current challenge with policy measures aimed at promoting sustainable tourism and enhancing Egypt’s competitiveness as a sustainable destination^17^. However, the adoption of SSCM in Egyptian hotels has been explored narrowly, particularly in the context of the varying responses by different hotel categories to sustainability pressures.

Recent studies have reviewed several recent publications that address shortcomings in the existing literature on the sustainability of hotel supply chains. However, most research to date has narrowly explored the adoption and motivation of environmental practices without examining their actual effects or the broader performance and stakeholder implications of hotel operations^18,19^. The social dimension of sustainability is often overlooked; however, it is essential to obtain a comprehensive understanding of sustainable supply chain management^20^. However, a large proportion of the literature also provides a specific focus on hotel types (independent, 5-star). It restricts their investigation to specific stakeholder groups (guests and employees), without addressing the roles of other supply chain members (such as suppliers and tour operators)^21–23^. This restricted view of complexity and linkages related to sustainability issues in the hotel industry is inappropriate. These limitations underscore the need for an overarching research model that encompasses environmental, social, and economic factors and considers all stakeholder groups involved in the hotel supply chain.

To guide our investigation, we draw on stakeholder theory, which provides a robust ethical and practical foundation for understanding how various groups influence and are influenced by sustainability practices^24,25^. While traditional SSCM research has often prioritized economic outcomes, stakeholder theory emphasizes the importance of balancing economic, social, and environmental goals^26,27^. This perspective is particularly relevant in the hospitality industry, where stakeholder expectations and pressures can vary significantly across hotel categories and operational contexts.

Additionally, there is little empirical evidence to support explanatory frameworks that associate SSCM practices with actual impacts on hotels and their stakeholders^28,29^. In doing so, the present study extends and fills the existing gap, employing a comprehensive stakeholder-based framework to explore the drivers, barriers, and impacts of SSCM in local and international hotel chains operating in Egypt. This method offers novel comparative perspectives and applies relevant guidance to the industry. To inform our inquiry, we employed stakeholder theory, as it provides a solid ethical and practical foundation for examining how different stakeholders shape, and are shaped by, sustainability practices^24,25^.

In contrast to earlier research, which typically isolates environmental initiatives or single stakeholder views, this study adopts a systemic perspective of the relationship between hotels and their suppliers by (1) taking a systematic perspective across three different hotel categories: international chains, 5-star local chains, and 4-star local chains, and (2) explicitly addressing the roles and perspectives of all of the most important supply chain stakeholders, including suppliers and tour operators. This model offers comprehensive insights into the drivers, barriers, and impacts of SSCM in Egypt’s hotel sector, where the economic significance of tourism and its environmental sensitivity are well recognized. With the multivariate qualitative design implemented, the research helps to fill the existing void in the literature regarding detailed, theory-informed analysis of the sustainability dimension in hospitality supply chains in less developed economies. This investigation is conceptually situated within the framework of stakeholder theory, which provides a robust ethical and practical foundation for understanding how sustainability practices impact various stakeholder groups^24,25^. Although the traditional SSCM literature tends to focus on economic performance, stakeholder theory emphasizes the importance of addressing not only economic factors but also social and environmental concerns as part of a strategic approach to achieving long-term business success^26,27^. In the hospitality industry, this equilibrium is particularly relevant, as stakeholder expectations and pressures can vary significantly between hotel types and operational contexts. Therefore, the following research questions (RQs) guided this study.

- RQ1. What types of sustainable supply chain practices (SSCPs) are implemented by Egyptian hotel firms?
- RQ2. Why do Egyptian hotels implement SSCPs?
- RQ3. What key barriers have hindered hotels’ adoption of SSCPs in Egypt?
- RQ4. Are there any differences or similarities among local 4-star chain hotels, local 5-star chain hotels, and international chain hotels in terms of SSCPs?

We reviewed the SHSCM literature and collected data on nine hotels operating in Egypt, employing a multi-case study research approach. Drawing on data collected using semi-structured interviews of local and international hotel chains, this study contributes to the literature by: (1) exploring the SSCPs drivers and barriers in a developing country from the hotel’s perspective, which is an under-explored area to date; (2) presenting SSCPs that span through the three dimensions of sustainability, and their impact on the environmental, social, economic, operational, and reputational performance of hotels; (3) performing a multi-level analysis of identified factors implementing SSCPs in supply chains (such as internal and external environmental practices; and organizational, supply chain, and community-level social or economic practices); (4) identifying the commonalities and differences between international chain hotels (ICHs), 5-star local chain hotels (LCHs5), and 4-star local chain hotels (LCHs4); and (5) developing a conceptual framework based on our empirical findings and stakeholder theory. The proposed framework can serve as a guiding paradigm for hotels, offering valuable insights on implementing sustainable practices.

The remainder of this paper is organized as follows. Section “Literature review” presents the background of this study. Section “Methodology” describes the research methodology. Section “Findings” presents the key findings, followed by section “ Discussion and conceptual framework”, which includes a discussion and conceptual framework. Section “Conclusion” concludes the study, outlining its limitations and providing directions for future research.

## Literature review

### Sustainable supply chain management

Sustainable Supply Chain Management (SSCM) is defined as [.] the management of material, information, and capital flows as well as cooperation among companies along the supply chain while taking goals from all three dimensions of sustainable development, i.e., economic, environment and social, into account which are derived from customer and stakeholder requirements[^30^, p. 1700].

Research in the field of sustainable supply chain management (SSCM) consistently demonstrates a strong causal relationship between the adoption of SSCM practices and the enhancement of SSCM performance. Numerous studies, such as Hussain et al.^11^, Kähkönen et al.^31^, and Wang et al.^32^ have provided compelling evidence that organizations embracing supply chain sustainability experience improved environmental, social, and economic outcomes. Firms can reduce their environmental footprint, enhance operational efficiency, lower costs, and strengthen their reputation by implementing green procurement, supplier collaboration for sustainability, human rights practices, and philanthropy initiatives.

#### Research gaps

Based on a review of the most relevant studies related to this study (Table ‎1), several common gaps between studies have been identified. The current research landscape on sustainable practices within the hotel supply chain reveals several interconnected gaps and limitations. Robin et al.^19^ focus on the adoption of environmental practices in independent hotels, probing into their drivers and barriers. However, their analysis stops short of examining the actual outcomes of these practices, thereby providing an incomplete assessment of their tangible impact. This gap in outcome analysis is somewhat echoed in the study by Abdou et al.^18^, which concentrates solely on eco-friendly 5-star hotels and their environmental sustainability, again neglecting broader implications. Cerchione and Bansal^20^ examined the impact of environmental practices on the environmental and economic performance of hotels. While valuable, their research overlooks the social aspect of sustainability, which is a critical component for a comprehensive understanding of sustainability in the hospitality sector. This oversight was similarly observed by Ibrahim et al.^23^, who, despite focusing on the social dimension, limited their study to a specific group (Greater Cairo hotel workers) and a single hotel category, thus missing a broader perspective.

Asadi^21^, also limit their scope to the impact of environmental practices. However, their study is narrowly confined to hotels and their guests, excluding other key stakeholders in the supply chain, such as tour operators and suppliers. This narrow focus, considering only the effects of environmental practices, overlooks other aspects of sustainability. This narrow lens is comparable to the approach of ElBelehy and Crispim^22^, which limits their study to the social aspects of five-star hotels and overlooks the multifaceted nature of sustainability once again.

Recent research has begun to explore the circular economy and its impact on hotel performance, with relevance in the MENA region. For instance, Zaki^16^ measured the effect of circular economy practices in eco-friendly hotels in Saudi Arabia and Egypt, finding that circular economy strategies (redesign, production, circulation, and recovery) positively influence hotel performance, and that Industry 4.0 innovations can augment this influence. However, Zaki^16^ primarily surveyed hotel employees and did not consider the views of other key stakeholders, the broader supply chain, the differences between categories of hotels, or the integration of social sustainability dimensions.

Khatter et al.^33^ identify barriers and drivers of environmentally sustainable practices in the Australian hotel industry but confine their exploration to environmental practices alone, not venturing into broader sustainability practices. Ghaderi et al.^34^ focus on the link between Corporate Social Responsibility (CSR) and financial performance, specifically examining hotels within the supply chain, thereby providing a limited view of CSR’s broader impacts. Peña-Miranda et al.^29^ highlight a significant gap in not examining the impact and outcomes of sustainable practices through effective stakeholder-related indicators. This oversight highlights the need for research that evaluates the effectiveness of sustainability initiatives in terms of stakeholder engagement. Alameeri et al.^28^, provide a theoretical framework for categorizing and prioritizing sustainable practices based on expert opinions; however, they do not delve into their practical impacts and outcomes, resulting in a lack of empirical validation and practical insights.

Together, these studies illustrate a fragmented approach to understanding sustainable practices in the hotel industry. They highlight the need for a more integrated research approach that not only considers a wider range of sustainability practices and their outcomes but also encompasses a broader set of stakeholders, including those beyond hotels and their guests, and extends to the entire supply chain. This comprehensive approach offers a holistic view of sustainability in the hospitality sector. This study surpasses previous work, which has predominantly focused on environmental aspects, by encompassing a broader spectrum of sustainability dimensions and comparing their application in both local and international hotel chains. Furthermore, by adopting a supply chain view, this study extends the scope of SSCPs research beyond considering their impact on hotel guests. The creation of an integrated conceptual framework that links SSCPs with their drivers, barriers, and consequent impacts provides a more comprehensive perspective on sustainability in the hotel supply chain, bridging gaps in the existing literature that often overlook these connections.

#### Initial conceptual framework

**Fig. 1:**
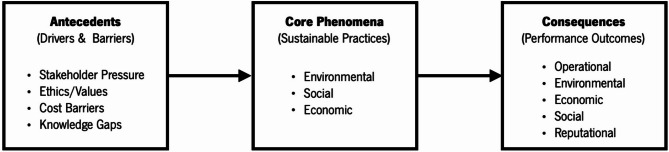
Initial Conceptual framework for sustainable supply chain practices in hotels.

Based on the integration of the literature and the theoretical foundation of the stakeholder theory, an Initial Conceptual Framework (Fig. 1) was developed for this study to guide data collection and analysis. This framework integrates insights from empirical studies conducted in different geographical and methodological contexts. For instance, drivers and barriers extraction is inspired by Alameeri et al.^28^; Khatter et al.^33^, who stress the role of internal motivations (e.g., ethics, cost savings, brand image) and external pressures (e.g., regulations, customer preferences) in explaining the adoption of sustainability. Similarly, the typology of sustainable practices captures the multiple dimensions of approaches used by ElBelehy et al.^22^, Ibrahim et al.^23^, and Peña-Miranda et al.^29^. The performance results were based on those reported by Zaki^16^, Asadi et al.^21^, and Ghaderi et al.^34^.

**Fig. 2:**
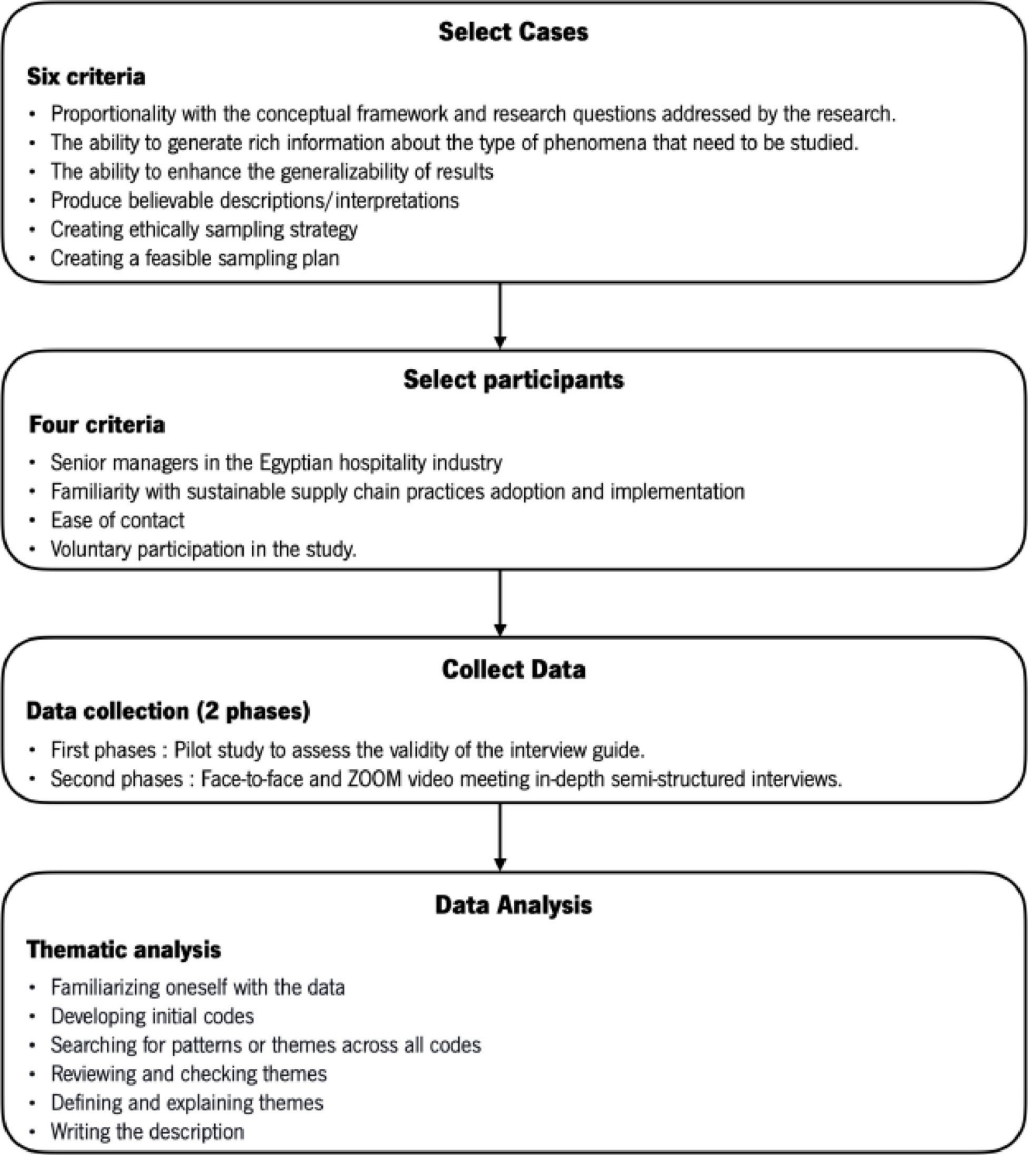
Overview of the methodology.

To address these deficiencies, the initial conceptual framework of the current study is based on three main dimensions: antecedents, core phenomena, and consequences.

- *Antecedents (Drivers and Barriers)*.

Antecedents encompass the drivers that motivate and the barriers that hinder the adoption of SSCPs in hotels. The key drivers identified include stakeholder pressure (from customers, regulators, and communities), ethical values, operational efficiency imperatives, and regulatory compliance. International hotel chains are subject to more intense stakeholder scrutiny and possess greater internal resources, facilitating the broader adoption of SSCPs. Conversely, barriers such as economic instability, knowledge deficits, limited financial resources, and contextual challenges, including those arising from Egypt’s post-revolution environment, impede the implementation of sustainable practices, especially in local hotels.

- *Core Phenomena (Sustainable Supply Chain Practices)*.

The central focus of this framework is the range of SSCPs adopted by hotels. These include responsible sourcing, waste reduction, energy and water efficiency, fair labor practices, and collaboration with suppliers and tour operators. The nature and level of these practices vary between hotel categories, with international chains generally having more advanced systems.

- *Consequences (Outcomes)*.

The use of SSCPs yields a range of results, from the obvious to the intangible. These include a better organizational reputation, more efficient resource utilization, cost savings, stronger customer loyalty, and increased stakeholder satisfaction. These results strengthen the business case for sustainability, supporting the long-term competitiveness of hotels.

### Sustainability practices in the hotel supply chain

#### Environmental

There has been growing emphasis on environmentally responsible management within the hotel industry, with green hotels emerging as a prominent model for sustainable development^35^. Given the inherent functions of hotels, which involve significant energy and water consumption, as well as the generation of substantial solid waste, pose environmental challenges^36^. The scope of environmental supply chain management in the service industry extends beyond internal functions and incorporates integration with both customers and suppliers^37^.

Sustainability, a defining feature in the hotel industry over the last decade, promotes the broader application of green practices, such as eliminating plastic disposal items, reducing unnecessary resource use, and minimizing food waste^38^. Furthermore, the government policy regulating this sector necessitates the incorporation of environmentally sustainable management within their supply chains^39^. Social activists, environmentalists, and government agencies are debating the global scenario for eco-friendly and sustainable hotels, putting pressure on businesses to change their overall business plans^38^.

To foster a sustainable and environmentally friendly supply chain, collaboration among all stakeholders is imperative^40^, (as green supply chain management works to organize interrelationships with all stakeholders^41^. The relevant stakeholders comprise the internal hotel supply chain, including managers, employees, owners, clients, customers, and suppliers^42^, as well as external parties such as government agencies, recycling and waste disposal firms, organizations supporting environmental protection, and charitable organizations^43^.

The customer-facing segment of the supply chain plays a critical role in advancing comprehensive sustainability objectives. Zaki^16^ investigated the extent to which hotels promote environmental collaboration, specifically by enabling and encouraging customer participation in sustainability initiatives. Espino-Rodríguez and Taha^44^ posit that customer integration is the reciprocal of supplier integration and hinges on proactively discerning and meeting customer requirements. Furthermore, adopting green practices sends a positive signal about a hotel’s environmental protection efforts, which can serve as a motivator for customers to identify with and associate themselves with the business^45^. This leads to customers spreading positive word-of-mouth about the hotel, enhancing customer awareness and engagement, and taking action to support employees in the green service process^46^. Additional measures, as suggested by Moise et al.^47^, include recycling and reusing, energy efficiency and conservation, water conservation, landscaping, and purchasing locally and ecologically produced goods.

#### Social

Social sustainability practices in the hotel supply chain can be classified into three categories: organizational level, supply chain level, and community level. The organizational level includes provisions for fair wages and benefits, maintaining a safe and healthy environment, employees’ development and training, employees’ welfare, equity, prohibition of child and forced labor, and regulatory responsibility^29,34,48,49^. The supply chain level includes practices of local purchasing to support the local economy and offering education and training to suppliers to improve sustainable awareness^29,49^. Finally, the community level refers to hotel investments in creating employment and business opportunities for the surrounding community, as well as providing education, training, and healthcare facilities, to establish the firm as progressive in the eyes of stakeholders^29,34,48,49^.

#### Economical

The economic practices of SSCM aim to achieve long-term profitability while minimizing negative environmental and social impacts^50^. Studies indicate that implementing environmentally and socially responsible practices is only viable when the economic stability of the enterprise is assured^51,52^.

Economic sustainability practices in the hotel supply chain can be classified into two categories: organizational level and supply chain level. The organizational level includes cost reduction and efficient resource utilization^20^; furthermore, the supply chain level includes long-term relationships with suppliers^53^. Hotels extensively use a wide variety of resources and energy, including air conditioning, lighting, heating, kitchen equipment, laundry, cleaning, as well as construction activities, catering, laundries, swimming pools, spas, and gardens^20^. Strategic resource conservation practices have become essential for both environmental preservation and improving the financial performance of hotels. Energy-efficient lighting systems, reusing linen and towels, composting food waste, using renewable energy sources, reducing resource quantities, rainwater collection, reusing water and building materials, buying reusable materials, and recycling initiatives are some of the key practices in this regard^54^. Cerchione and Bansal^20^ highlighted that there is a positive association between hotels’ financial performance and resource conservation. On the other hand, neglecting to implement sustainable resource usage habits might cause irreversible harm to the ecosystem, endangering economic prosperity^55^.

To achieve better environmental and financial results, Asadi et al.^21^ emphasize the need for hotels to adopt clean and energy-efficient technology and equipment. They specifically call for lowering energy usage in lighting and air conditioning systems. Furthermore, Cingoski and Petrevska^56^ contend that energy-efficient efforts boost the hotel’s overall aesthetic reputation, lower operating expenses and maintenance system failures, increase profitability, and strengthen its position in the market, in addition to ensuring guest comfort.

#### Drivers and barriers

According to existing literature, an organization may be compelled to adopt sustainable practices in the supply chain due to various factors that can be categorized as either internal or external drivers. The key drivers in the adoption of SSCPs are pressures from stakeholders^57^, customers^33^, the government^33,58^, employees^33^, investors^59^, and competitors^60^. In contrast, studies suggest that for hotels, the primary drivers for SSCM adoption are internal factors, such as financial reasons, operational efficiency, ethics and values, and management commitment^21,33,58,61,62^ and the government regulations or legislation^21,33,58,61,62^ .

Barriers are impediments that can hinder or prevent the implementation of sustainable supply chain management practices^63^. They can be classified into internal and external factors^64^. External barriers to sustainability include the absence of government and industry regulations^65,66^, as well as competitive pressures^67^. Furthermore, a lack of understanding and awareness can hinder the adoption of SSCPs^65,66^. Hotels also face various barriers to sustainability, such as resource unavailability, high costs of sustainable practices, and resistance to adopting sustainable innovations^33,65,66^.

### Stakeholder theory in the sustainable hotels’ supply chain

Stakeholder theory (ST), as formulated by Freeman^68^, analyzes the various individuals or parties—stakeholders—and their connections with organizations, which may influence business operations. These organizations may also feel that they are affected by the way society reacts to them in return. In hotels, when applied to sustainable supply chain management (SSCM), it offers a way of understanding why differing stakeholder pressures have so shaped both the adoption and implementation of sustainability practices^57^.

Our findings suggest that stakeholder theory is a key factor in explaining why hotels in Egypt adopt sustainable practices. For example, international hotel chains respond to global customer expectations and international standards by adopting advanced sustainability initiatives. In contrast, community needs, supplier relationships, and local regulations have a greater influence on local chains. This argument is validated from the ST point of view that the distribution of value and criticism should come more broadly than simply to shareholders. Who are the critics—employees, suppliers, customers, the government, NGOs, and the local community^57^?

Stakeholder pressures—both internal and external—play a decisive role in influencing a hotel organization to raise awareness of sustainability and subsequently implement it^69^. In our study, we find that a blend exists between ethical pressures (normative motives) and an incentive to increase name or operational efficiencies, which together shape hotels’ sustainability efforts. This has been mentioned in prior research^70,71^. Furthermore, stakeholders have varying impacts on hotels and their supply chain links, a point that has been observed in numerous studies^70,72^.

### Outcomes of sustainable supply chain practices

The outcomes of sustainable supply chain management refer to the performance of supply chain practices within firms and encompass all sustainable dimensions of the triple bottom line — economic, environmental, and social^73,74^. Succeeding research endeavors have augmented the scope of outcomes by introducing two additional categories: operational outcomes^75^ and public image and reputation^76^. Companies that implement SSCM generally outperform their competitors in terms of economic performance and have reported advantages, including increased productivity, higher employee retention, fewer operational errors, and fewer accidents^77,78^. Specific results, such as improving reputation and gaining market share, meet the original motivator to adopt sustainable practices^76^.

Green supply chain management is one of the most effective strategies for reducing waste, pollution, and environmental degradation, as it has a significant impact on the environment^79,80^. Specific practices have been linked to positive hotel environmental performance: energy use reduction^11^, water use reduction^81^, waste reduction, reduction of carbon emissions, recycling, and reuse^80^, and green purchasing^82^. However, rather than preserving the environment, most environmental investments made in developing countries are meant to save operating costs.

By adopting SSCM practices, firms can enhance their capabilities and outperform competitors in terms of environmental efficiency and social responsibility. There are positive outcomes associated with social initiatives. For example, providing employees with training opportunities has a positive impact on their job satisfaction and self-confidence^53^. Furthermore, the corporate social responsibility practices of hotels influence the local community by strengthening altruistic intentions, such as promoting societal well-being and environmental protection^83^.

Some argue that investments in sustainability create exceptional value for shareholders^84^, which has a direct impact on the firm’s economic outcomes^85^. Chen andChen^53^ argue that the installation of energy-saving and water-efficient technologies or equipment in hotels leads to saving costs and Improved relationships with stakeholders, partners, and networks. The conservation of used resources has a positive impact on hotels’ financial performance^86^. Furthermore, energy-efficient initiatives can reduce the failures of maintenance systems and operating costs, improve profits, and impose market competitiveness^56^.

In terms of operational performance, firms have been incorporating sustainability into business performance measures^87^. Those that implement sustainable initiatives can enhance their operational efficiency and financial performance by reducing related economic, social, environmental, and political costs, and increasing access to critical resources^86,88^. Moreover, corporate social responsibility initiatives related to the quality of work-life have led to job satisfaction^89^.

## Methodology

### Research setting

Egypt has been selected as the context for the current study to make a contextual contribution, as the country’s economy has grown to be the second largest in Africa, the sixth largest in the Middle East and North Africa (MENA), and the 36th largest in the world^90^. Moreover, Egypt’s economy is among the most diverse in the MENA region, encompassing farming, industry, tourism, and services. Egypt’s GDP (gross domestic product) has nearly doubled from $20 billion in 1960 to $412 billion in 2020^88,90^. Statista^91^ reports that the hospitality and tourism industry contributed approximately $ 12.6 billion to the Egyptian economy in 2018.

Furthermore, the industry employed 2.16 million people in 2021. It is expected that this sector will contribute 19.5 billion USD to the Egyptian economy by 2028^92^. Furthermore, to promote social responsibility, the Egyptian government has implemented regulations and laws that provide incentives for donations and subsidies to various entities, including associations, public educational institutions, public hospitals, and scientific research institutions. According to the Presidency of the Arab Republic of Egypt^93^, these donations and subsidies are tax-deductible from the firm’s net profit if they amount to less than 10% of the net profit. However, Egypt ranks 127th out of 180 nations in the 2022 Environmental Performance Index, and its environmental performance in terms of Climate Change, Environmental Health, and Ecosystem Vitality is lower than that of its neighboring MENA nations^94^. Furthermore, approximately 61% of industrial and investment companies in Egypt do not contribute to supporting activities in any corporate social responsibility area^95^. These findings underscore the compelling need for research in the field of sustainability in the Egyptian context.

### Research design

This research employed a qualitative, exploratory, multi-case study design, an approach that examines one or more instances of social phenomena, gathering large amounts of detailed data from these cases^96^. Multiple case study is the appropriate method when there is contemporaneity of the content and it is impossible to manipulate the behaviors^97^, as in our study. Furthermore, following the recommendations of Cooper et al.^98^, a case study was deemed appropriate because it necessitated detailed and intensive analyses to identify issues and generate insights that would make a contextual contribution. Case studies have already been widely used as a method to explain social and environmental practices in several sectors^99^. Furthermore, Robin et al.^19^, Pereira et al.^100^, Chen and Chen^101^ have used case studies as the basis for research on the social and environmental development of hotels.

This study aims to develop an integrated conceptual framework that connects sustainable supply chain practices (SSCPs) with their drivers, barriers, and outcomes. Stakeholder theory is used to explain the motivations behind implementing SSCPs. The research also examines how these factors vary or remain consistent across different hotel categories and explores the adoption of sustainable initiatives based on both normative and instrumental logic. This approach aligns with theory elaboration, which involves contextualizing a general theory and designing empirical research using existing conceptual models as a foundation^102^.

To answer the research questions, we conducted case studies within the Egyptian hotel industry. The case study methodology is an empirical approach suitable for improving our understanding of “real-world” events through an in-depth examination of a current phenomenon over which the researcher has little control^97^. According to Yin^97^, the evidence resulting from multiple-case studies is considered more convincing and yields more robust global results. This study employed an exploratory, multiple-case strategy (see, e.g.^103,104^, to examine sustainable practices in the hotel supply chain. The lack of literature combining the three dimensions of sustainability in the hotel supply chain (see, e.g.^105,106^, highlights the need to consider possible interactions between environmental, economic, and social sustainability practices to generate financial, environmental, and social value for stakeholders^107,108^. A multiple-case strategy allowed us to empirically analyze the sustainable practices of several cases in a fine-grained, in-depth, and contextualized way, as well as compare cases and map the SSCM of hotels in a developing country. Figure ‎2 presents an overview of the methodology.

We selected nine cases according to the six criteria for cases selection by Miles and Huberman^104^ that prescribe that the sampling strategy should (1) be relevant to the conceptual framework and the research questions addressed by the research; (2) be likely to generate rich information on the type of phenomena which need to be studied; (3) enhance the generalizability of the findings; (4) produce believable descriptions or explanations; be ethical, and be feasible. Indeed, in a multiple-case study design, some degree of generalizability can only be obtained within the context in which the study was conducted.

The sample includes Egyptian hotels of different categories (local chain 4-star hotels, local chain 5-star hotels, and international chain hotels), and from four different regions: Aswan, Luxor, the Red Sea, and Cairo. While the sample size is limited, the selection strategy ensures that the most relevant and diverse perspectives within the Egyptian hotel sector are included. The focus on major hotel categories and regional diversity allows the findings to reflect key trends and challenges in the industry. However, as with most qualitative research, the results are intended to provide in-depth, context-specific insights rather than statistical generalizations^98^.

### Participant selection and data collection

The case studies are hotels of varying types (3 local chain 4-star, three local chain 5-star, and three international chain 5-star) to gather data between August 2020 and December 2022. Purposive sampling^109^ was implemented to select the participants guided by the four conditions proposed by Soundararajan and Brown^110^: (1) The involvement and pertinence of informants to the research context were assessed, leading to the establishment of the inclusion criterion “being manager at hotel in Egypt”; (2) their understanding about the adoption and execution of sustainability practices; (3) accessibility for communication of contact, and (4) willingness to voluntarily participate in the study. We conducted in-depth, face-to-face and Zoom video-conferencing semi-structured interviews with 2 participants in the pilot study and 18 in the main study. All participants are hotel managers in Egypt. Information about the selected hotels and interviewed participants is presented in Table ‎2.

Ethical approval for this study, including the experimental protocol, was obtained from the College of International Transport and Logistics Research Ethics Committee, Arab Academy for Science, Technology & Maritime Transport, on 10 July 2020. The research was conducted in accordance with the ethical guidelines of the Egyptian Ministry of Higher Education and Scientific Research, which regulate ethical research involving human participants in non-medical fields. Furthermore, the study adhered to general ethical principles outlined in international research integrity frameworks.

The pilot study involved two managers, one each from a local chain hotel and another from an international chain hotel, to assess the validity of the interview guide. A face-to-face interview was conducted with the first, and one via ZOOM with the latter. In response to the feedback received during the pilot study, it was deemed necessary to refine the interview protocol questions. Hence, four questions, evenly split between environmental and social practices, were modified to broaden the investigation beyond the focal hotel and to encompass supply chain members. The participants in the pre-study were not included in the main study.

In the second phase (main study), two managers in each of the nine hotels analyzed were interviewed, in a total of 18 interviews (10 face-to-face and eight via ZOOM), 6 with managers from local chain 4-star hotels, six interviews with managers from local chain 5-star hotels, and 6 with managers from international chain hotels. The use of ZOOM was necessary due to the geographically dispersed location of the interviewees. All interviews were conducted in Arabic, recorded with the participant’s consent, and subsequently transcribed and translated into English for data analysis, resulting in 99 pages of transcripts. The average duration of the interviews was 45 min.

To enhance reliability^111,112^, a constant semi-structured interview guide was employed. Additionally, we maintained a record-keeping system for interviews and field notes to enhance the reliability of our findings^64^. The interview questions (refer to Appendix A) were derived from the SSCM literature, as detailed below, to ensure a solid rationale; however, issues were reformulated in language suitable for our interlocutors to understand. The interview guide includes an introduction and objectives of the study, general guidelines, questions about demographic information, and questions on the research topic that cover four themes:

1. SSCPs, including environmental, social, and economic aspects^19,61,113^;
2. Drivers sought to explore the motivating factors behind the hotel’s adoption of sustainable practices^64,114^;
3. Barriers identifying the challenges faced by hotels^33,64^.
4. Outcomes, aimed to gain an understanding of the benefits acquired through the application of the practices^19,63^.

Some of the questions are formulated in a precise manner, seeking a concrete answer, while others are more open-ended, encouraging the interlocutor to respond more freely. Depending on the course of the interview, new questions could arise to clarify and specify some of the answers.

Among the 31 interview questions, six specifically focus on evaluating the hotel’s environmental sustainability practices regarding its supply chain. These questions cover a range of topics, including organizational procedures related to environmental considerations in supplier selection, product purchasing, reverse logistics, and waste management, as well as initiatives aimed at reducing energy, water, and food consumption. The questions further explore monitoring processes for resource usage, collaborations with suppliers and tour operators to promote environmental improvement, and the hotel’s engagement with government agencies to ensure environmental compliance and support local sustainability efforts.

In parallel, of the 31 interview questions, nine are specifically tailored to assess social sustainability practices. These questions assess the organization’s commitment to employee development and welfare, as well as its initiatives promoting equity and supporting women, raising awareness of child and forced labor in the supply chain, and implementing formal procedures related to labor practices, human rights, and fair wages. Additionally, the questions delve into education and training for suppliers, collaboration on health and safety standards, preferences for local sourcing, and practices supporting local communities in Egypt.

Furthermore, within the set of 31 interview questions, three are dedicated to exploring economic sustainability practices. These inquiries center around the strategies employed for cost reduction without compromising service quality, specific examples of successful cost-saving initiatives, and the organization’s practices related to maintaining long-term purchasing relationships. The focus is on evaluating the hotel’s economic efficiency, cost management strategies, and commitment to fostering enduring relationships with suppliers.

In a related vein, out of the 31 interview questions, seven are specifically designed to uncover the drivers behind the hotel’s implementation of sustainable practices in its supply chain. These questions probe the rationale behind each environmental practice, the advantages gained, the influencers shaping the organization’s environmental practices, and the pursuit of green certifications. Additionally, the questions extend this inquiry to social practices, exploring influences, motivations, and reasons behind the creation of social programs and initiatives within the organization.

Lastly, among the 31 interview questions, five are dedicated to exploring the barriers that hinder the hotel’s implementation of sustainable practices in the supply chain. These questions investigate the difficulties encountered in applying both environmental and social practices, the disadvantages associated with their application, and the specific challenges faced in implementing such practices.

Data saturation was achieved through an iterative process of data collection and thematic analysis, continuing until no new themes or significant insights emerged. Specifically, after the seventh interview, recurring patterns and themes became evident, and by the 13th interview, theoretical saturation was reached, with 48 different codes applied. The final five interviews confirmed that no new themes, concepts, properties, or dimensions were uncovered. This approach aligns with established qualitative research standards, which define saturation as the point where additional data no longer yields new codes or themes. Recent literature suggests that saturation in studies with homogeneous objectives and populations is often reached within 9–17 interviews, supporting the adequacy of the sample size used in this research^115,116^.

Our research involved face-to-face interviews and onsite observations in five of the nine hotels selected. The hotels we have visited are ICHs.1, LCHs5.1, LCHs5.2, LCHs4.1, and LCHs4.2. Through on-site visits, one can observe firsthand how the hotels engage in sustainable practices. The ICH’s one hotel has been the most active in promoting various aspects of environmental sustainability. There were numerous waste separation bins in the guest areas, an obvious use of eco-friendly cleaning products, and a wide adoption of energy-saving lighting. Certificates for environmental work and ISO certifications were displayed in the hotel lobby, as well as in other public spaces, attesting to its commitment to sustainable standards. A careful reading of the employee handbook revealed sensitive information related to labor practices and ethics, demonstrating the hotel’s serious commitment to fair work. Its promotional materials also highlighted its sustainability initiatives. In this way, the guest would understand the extent of the hotel’s environmental responsibility. The first local 5-star hotel (LCHs5.1) was also unique in our sample as it incorporated pieces of craftsmanship as integrated elements into its interior design. The second local 5-star hotel (LCHs5.2), which features a waste and sewage management system incorporating composting, is an ecological option. Two local 4-star hotels (LCHs4.1 and LCHs4.2) were also noticeably geared toward sustainability, with LED lighting and significant evidence of sourcing locally produced goods. For the remaining four hotels (ICHs 2, ICHs 3, LCHs 5.3, and LCHs 4.3), we opted for Zoom meetings due to geographical distances. Despite not physically visiting these establishments, the virtual interviews facilitated a comprehensive exploration of their sustainable initiatives, ensuring a holistic representation of diverse practices across the entire sample.

### Data analysis

We deployed the thematic analysis technique^117^ to code and analyze the interview data. First, we transcribed all interviews word for word, read them repeatedly to ensure familiarity with the content, and noted early ideas. Next, we developed the initial codes from the transcribed interviews. An inductive approach was adopted to generate all codes, themes, and dimensions from the interview data, which means that the codes and themes derive more from the concepts and ideas the researcher brings to the data during analysis^118^. We adopted a similar process to develop the second-order themes and aggregate themes, which involved collating the initial codes to form the next-order themes based on comparable topics and assembling them to generate main themes related to SSCPs, drivers, and barriers. Table ‎3 provides the step-by-step process of thematic analysis adopted in this study.

The collected data were analyzed with ATLAS.ti 23, a computer-aided qualitative data analysis software program. ATLAS.ti 23 software assisted in organizing the interview transcripts to develop the analysis. This tool enabled us to systematically code and analyze the data, including counting the frequency of codes mentioned in the passages, and to efficiently identify and compare codes across the different categories of hotels (local chain 4-star hotels, local chain 5-star hotels, and international chain hotels). Furthermore, ATLAS.ti 23 helped to ensure the accuracy and consistency of our analysis, as we were able to easily track and compare codes across the different types of hotels.

**Fig. 3:**
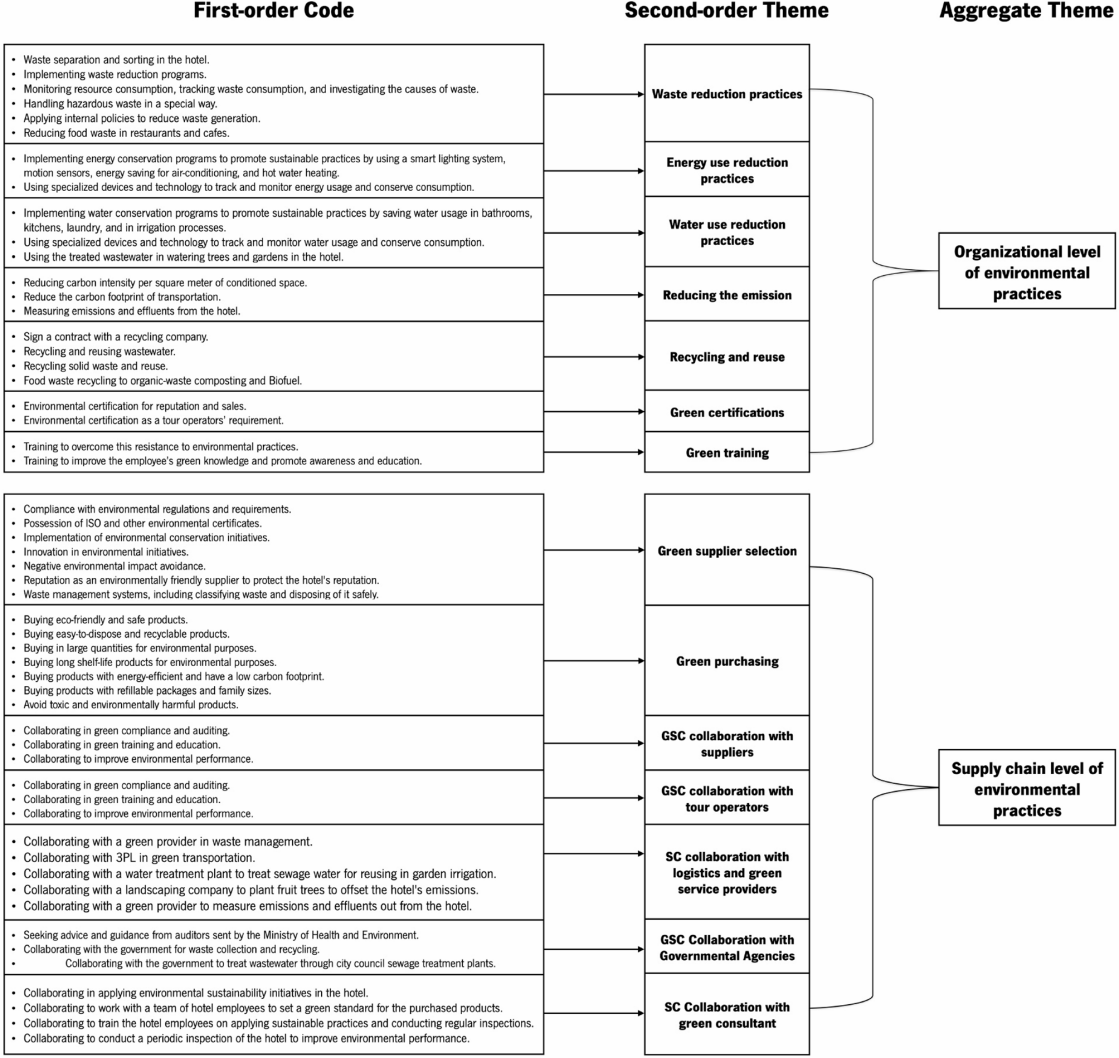
Coding structure of aggregate dimensions for environmental pratices derived from interviews content.

The data analysis process generated two aggregate themes related to Environmental Sustainability Practices (SPs) for the hotels (at the company or supply chain level), three aggregate themes for Social SPs (organizational, supply chain, and community levels), two aggregate themes for Economic SPs (organizational and supply chain), and two aggregate themes for drivers and barriers (internal and external). The analytical coding method for deriving aggregate themes is illustrated in Figs. 3 (Environmental SPs), 4 (Social SPs), 5 (Economic SPs), 6 (drivers), and 7 (barriers). We further analyzed the drivers in consideration of stakeholder theory. A central feature of qualitative research is the removal of doubts regarding the validity and reliability of the results^64^. We ensured validity by corroborating the findings through multiple sources of evidence^111^. We triangulated data through site visits, verbatim comments, and careful attention to various explanations related to sustainability across hotels^111^.

**Fig. 4:**
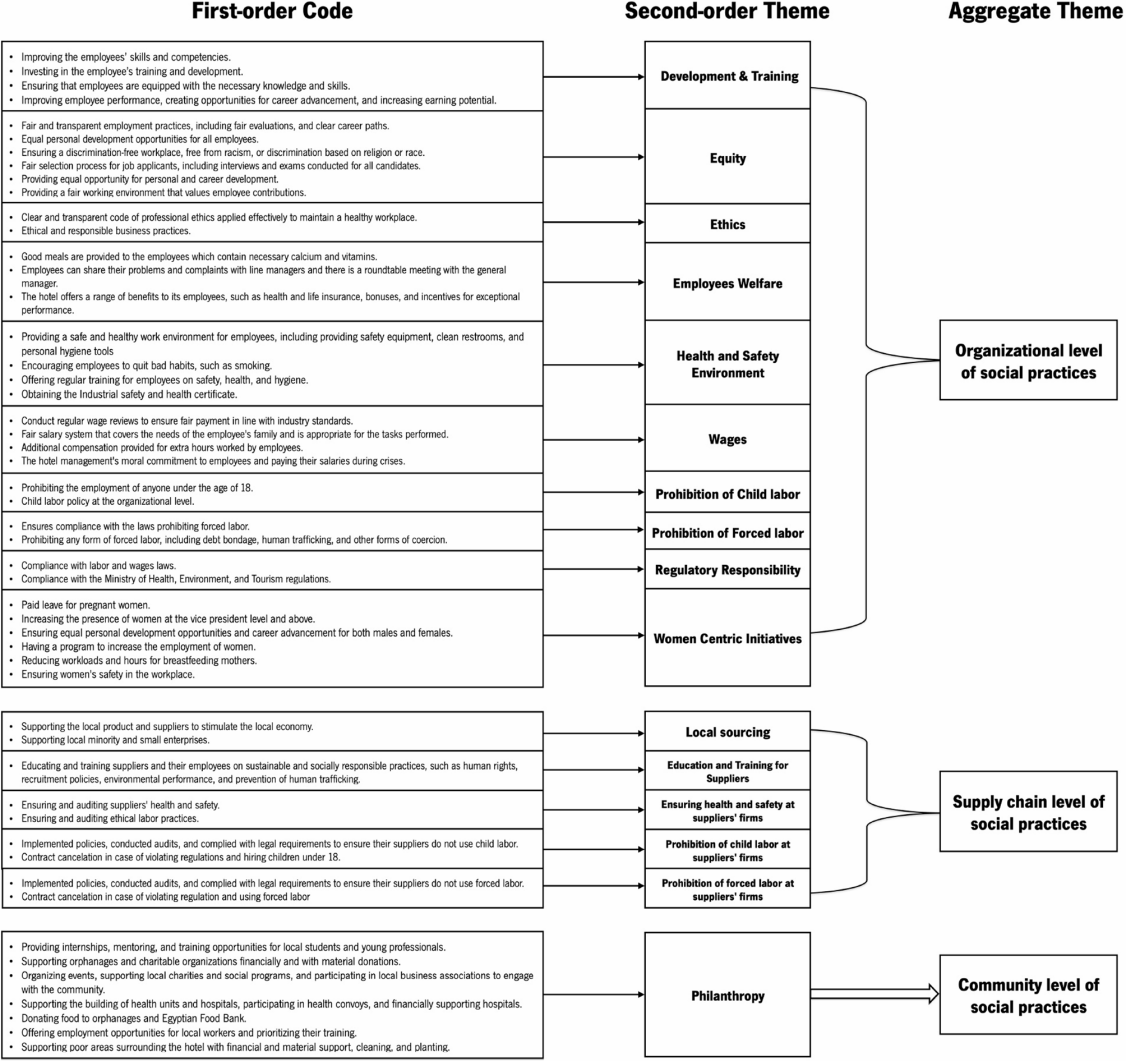
Coding structure of aggregate dimensions for social pratices derived from interviews content.

## Findings

The following sub-sections discuss the similarities and differences among 4-star local chain hotels (LCHs4), 5-star local chain hotels (LCHs5), and international chain hotels (ICHs) associated with SSCPs and their drivers and barriers (see Tables 4, 5, 6, 7 and ‎8).

### Environmental sustainability practices

Egyptian hotels implement environmental practices related to waste reduction, energy use reduction, and water use reduction; reducing carbon emission; recycling, and reuse; green certifications; green training; green supplier selection; green purchasing; green collaboration with suppliers; green collaboration with tours operators; collaboration with logistics and green providers; green collaboration with government, and collaboration with a green consultant (See Fig. 3). Our exploration of environmental practices encompasses the coding structure of aggregate dimensions for environmental practices employed by hotels. Table 4 summarizes the number of mentions of ecological sustainability practices by the managers of case hotels.

#### 4-star local chain hotels

The LCHs4 in the sample prioritize waste, water use, and energy use reduction as their Organizational Environmental SPs: *“We monitor the consumption of energy*,* water*,* and food*,* and we always strive to reduce waste and preserve the environment.”* (General Director, LCHs4*2). Two of the three LCHs also implement recycling and reuse initiatives, including collaborating with recyclers who collect their waste and recycle it, as well as supporting corporations and NGOs. All three hotels prioritize green certifications to enhance their reputation and present to tour operators.

Regarding the supply chain level of environmental practices, the LCHs4 prioritize Environmental SPs related to selecting suppliers based on their ecological characteristics. This was mentioned 12 times by the three LCHs4. As noted by a purchasing director, *“A committee composed of members of the hotel’s purchasing department also makes a field visit to the supplier’s factories to make sure the environment reduces resource consumption.”* (Purchasing Director, LCHs4*3).

Green product purchasing is also essential, mentioned 10 times by the hotel managers. The hotels also collaborate with tour operators, offering green services and amenities to promote long-term contracts: *“In the beginning*,* one of the tour operators sent a group of trainers to the hotel to train employees on how to implement environmental practices and how to preserve the environment by training them on how to reduce resource and energy consumption and others.”* (Financial Director, LCHs4*1). Collaboration with logistics and green service providers was mentioned six times, where specialized companies are contracted to manage waste disposal. For example, *“We contract with a specialized company in waste management that collects waste from the hotel*,* processes it*,* recycles it*,* and safely disposes of some of it.”* (Purchasing Director, LCHs4*2). Two of the three hotels (LCHs4*1 and LCHs4*2) also highlight the importance of consulting with green consultants to improve the quality of their Environmental SPs.

#### 5-star local chain hotels

LCHs5 prioritize waste reduction practices (mentioned 23 times by the interviewed managers). Regular check-ups and awareness programs are also in place. In addition to reducing water use, these hotels prioritize recycling and reuse to conserve resources. Three LCHs5 monitor energy consumption to reduce usage (mentioned 5 times). Holding a green certification is also emphasized to enhance public image: *“One of the main reasons for the hotel’s focus on obtaining environmental certificates and implementing environmental initiatives was to increase its sales and improve its image among tour operators.”* (General Director, LCHs5*1). Regarding green training in hotel operations, the hotels overcome the employees’ resistance to applying Environmental SPs by training and introducing their importance to the hotel and community:

*“One of the most important things that we are currently focusing on is environmental procedures. For this reason*,* the hotel has contracted with a consulting company specializing in sustainability to train the hotel employees on how to apply sustainable practices and to conduct regular inspections twice a month.”* (General Director, LCHs5*2).

Regarding the supply chain level of Environmental SPs, the LCHs5 prioritize green supplier selection practices (17 mentions). The interviewees also emphasized the importance of applying green supplier selection practices, as environmentally responsible suppliers are a crucial step in achieving a sustainable supply chain and enhancing the hotel’s image among guests. Purchasing green products is also highly valued (11 mentions): *“We have a set of requirements that must be available in the product before making a purchase*,* and one of the most important of these requirements is that these products should not contain any harmful materials to the environment and should be environmentally friendly.”* (General Director, LCHs5*3).

Furthermore, LCHs5 prioritize supply chain collaboration for environmental sustainability (green partnership with tour operators was mentioned 11 times, while the logistics and green providers 9 times, and green consultant collaboration 6 times): *“The hotel agreed with a consulting firm that specializes in sustainability research*,* and together they developed a strategy to transform the hotel into a sustainable establishment.”* (Purchasing Director, LCHs5*2). Only one hotel (LCHs5*1) has a green collaboration with government agencies; this hotel also collaborated with suppliers to improve environmental performance, reflected in the hotel’s performance: *“The hotel provides necessary advice and guidance to suppliers to improve their environmental performance.”* (General Director, LCHs5*1).

#### International chain hotels

ICHs share similar environmental SPs with LCHs 4 and LCHs 5, focusing on waste reduction (32 mentions). They prioritized energy reduction using LED lighting, motion sensors, and usage monitoring (12 mentions). Recycling and reuse are also important, with contracts for solid and food waste being repurposed for composting and biofuel production. Water conservation is prioritized using metering tools to evaluate consumption and implement day-to-day practices that conserve water.

*“We have a module in our sustainability program called food waste management*,* and one of its operations is to evaluate the waste of restaurants and cafes and estimate its value to sell it to the companies that use it in producing organic waste composting.”* (Sustainable Program Director, ICH.1).

Interviewees from ICHs expressed a strong interest in reducing carbon emissions (12 mentions), working with specialized companies to measure and minimize them: *“As part of our 2025 Sustainability and Social Impact Goals*,* our hotel aims to reduce carbon intensity per square meter of conditioned space by 30% from a 2016 baseline.”* (Marketing and Sales Director, ICH.3). Obtaining green certification for marketing purposes is also emphasized.

ICHs prioritize green suppliers (37 mentions) to reduce carbon emissions, preserve the environment, and attract environmentally conscious guests. During the interviews, it was mentioned (in 11 instances) that the hotels place great importance on purchasing green products, aiming to buy eco-friendly and safe products that are easy to dispose of and recycle. They also buy in large quantities and purchase long shelf-life products to reduce environmental waste. The ICHs value environmental supply chain collaboration as they believe that sustainability cannot be achieved through their efforts alone. They recognize the importance of working with partners such as suppliers, tour operators, logistics and green providers, green consultants, and government agencies to promote Environmental SPs: *“We have contracted with the Ministry of Petroleum to collect used oils and recycle them into soap.”* (Purchasing Director, ICH.2). However, only one hotel representative (ICH.2) mentioned collaborating with a green consultant during interviews.

### Social sustainability practices

The analyzed hotels implement different types of Social SPs which can be categorized in the following distinct categories: (1) practices at organizational level, such as development and training, equity, ethics, employees welfare, maintaining a healthy and safe environment, fair wages, prohibition of child labor, prohibition of forced labor, regulatory responsibility, and women-centric initiatives; (2) practices at supply chain level, such as local sourcing; education and training for suppliers; ensuring suppliers health and safety conditions; prohibited practices of child and forced labor for suppliers; and finally, (3) practices at community level, like philanthropy initiatives (See Fig. 4). Our exploration of social practices delineated the coding structure of aggregate dimensions for social practices by the hotels. Table 5 summarizes the number of mentions of social sustainability practices by the managers of case hotels.

**Table 1:** Recent contributions in the field of sustainable practices in the hotel supply chain.

Citation	Region of research	Aim	Methods	Main results	Limitations	Future research
Robin et al.^19^	Spain & Chile	Examine the adoption of environmental practices in the hotel industry and its impact on independent hotels in mature and emerging destinations. The study focuses on two destinations, Madrid in Spain and Santiago and Valparaiso in Chile, and aims to compare the implementation of environmental practices in these two contexts	A qualitative method based on case studies of 24 hotels, data collected through Semi-structured interviews	The adoption of environmental practices in both surveyed destinations elucidates the multifaceted consequences of this implementation, primarily in the financial and operational spheres. Differences are observed in the two countries regarding the proposed model, mainly in terms of barriers to implementing environmental practices, products used, and processes related to clients’ and suppliers’ responsibilities	The interviewees were not hotel guests but senior managers, so it was not possible to obtain a broader picture of guests’ perceptions of sustainable practices	- Future research could focus on direct customer perceptions and evaluations related to the sustainable practices offered by independent hotels. - Future research can conduct a quantitative study with a representative sample of independent hotels through sampling techniques
Alameeri et al.^28^	UAE	Developing a framework to identify, categorize, and prioritize sustainable management practices in the UAE hotel sector	Mixed methods (qualitative and quantitative): an extensive literature review and expert opinions collected through in-depth interviews using: Analytical Hierarchy Process (AHP), involving numerical scoring and ranking based on a structured survey	Employee management and government management take top priority under the main criteria, and policy requirements, customer culture, and education and training were determined to be the three most relevant sub-criteria for sustainable practices for hotels in the UAE. Furthermore, the hotels mostly focus on economic sustainability; however, the environmental and social dimensions of sustainability are ignored in management practices	The data was collected from only four and five-star hotels in the UAE	Future research can use the sustainability model of this research to measure sustainability in different hotel categories, further, the model can be used in different sectors
Ghaderi et al.^34^	Iran	Investigating and illuminating critical aspects of the relationship between corporate social responsibility and the performance of four and five-star hotels, focusing on the Iranian capital, Tehran	Quantitative approach: questionnaire survey of 350 employees of hotels; data analyzed using structured equation modeling	CSR initiatives positively on hotels’ performance within the context of four and five-star hotels in Tehran. These effects apply to all the five dimensions of CSR (social, economic, legal, ethical, and environmental)	The data was collected from small number of hotels in Tehran, the majority under a mix of public and private ownership and operation	Future research could focus on different types of hotels in other areas and explore the processes by which CSR impacts financial performance and relevant mediators
Cerchione and Bansal^20^	India	Exploring the impact of environmental policy and training aspects on hotels’ sustainability practices, along with evaluating the consequential effects of these practices on both environmental and financial performance	Quantitative approach: questionnaire survey of 312 managers from the Indian hospitality industry; data analysis using structural equation modeling	The environmental policy and training positively impact environmental communication, resource preservation, and energy preservation in the Indian hotel sector	The research focused only on the environmental dimension, with the absence of other social and economic dimensions	Future research could focus on the managerial alignment of social perspective with environmental and financial perspectives
Asadi et al.^21^	Malaysia	Exploring the impact of green innovation on the association of environmental, social, and economic aspects of sustainable performance, and Evaluating the outcomes based on the experiences of a selected sample of Malaysian hotels	Quantitative approach: questionnaire survey from 183 hotels in Malaysia; data was analyzed using structural equation modeling	The study shows that green innovation is positively associated with the environmental and social performance of the business, and economic performance is another important influencing factor	The research focused only on the effect of green practices on the sustainable performance of hotels	Investigate other factors related to CSR or the Economic dimension of sustainability that influence the sustainable performance of the same industry
Khatter et al.^33^	Australia	Exploring the barriers to and drivers of environmentally sustainable practices in the Australian hotel from the perspective of hotel managers	Qualitative research approach involving semi-structured in-depth interviews with 8 hotel managers	The major barriers to adapting environmental practices are time, financial challenges, availability of resources, and the views and imperatives of hotel owners and shareholders. The major drivers are financial, marketing, owner, and shareholder interests, and guest preferences	The research data were collected from the hotel managers, so it represents only the perspective of hotel managers	Obtain the views of other hotel stakeholders (i.e., suppliers, guests, employees) to gain a deeper understanding of the barriers to and drivers of environmental practices
Peña-Miranda et al.^29^	Colombia	Exploring the sustainable practices in the Colombian hotel industry	Qualitative method based on case studies of 8 hotels, data collected through Semi-structured interviews	The sustainable practices of Colombian hotels are categorized into three classes: philanthropic-reactive, legal-reactive, and active groups	- The research only considered eight hotels in Santa Marta, Colombia - The study primarily relied on data collected from hotel managers, which did not include a comprehensive analysis of all stakeholders involved in sustainable practices - The study did not provide a quantitative analysis of the outcomes or impacts of the sustainable practices observed in the hotels	- Examine the opinions of other hotel stakeholders, such as employees, guests, and the local community, to establish a more comprehensive understanding of sustainable practices in the hospitality industry - Explore the impact and outcomes of sustainable activities by designing and implementing indicators that have a direct relationship with stakeholders. This would help in measuring the results of sustainable initiatives effectively
Abdou et al.^18^	Egypt	Investigating the impact of environmentally sustainable practices (ESPs) on green satisfaction (GS) and customer citizenship behavior (CCB) in five-star hotels in Egypt and explore the potential mediative role of GS in the nexus between CCB and ESPs	Quantitative approach: questionnaire survey from 437 customers of hotels in Egypt; data was analyzed using structural equation modeling	ESPs significantly positively affect both GS and CCB. GS significantly impacts CCB, and GS partially mediates the relationship between CCB and ESPs	Focus on a specific customer demographic (Egyptian customers in five-star eco-friendly hotels)	Expand the scope of research to include other customer demographics and investigate the role of ESPs in different types of hotels
Ibrahim et al.^23^	Egypt	Exploring the direct and indirect effects of perceived CSR on employees’ engagement through organizational identification in the hotel industry	Quantitative approach: questionnaire survey from 420 employee in Egypt; data was analyzed using structural equation modeling	CSR practices and identification are crucial drivers of employees’ engagement; CSR toward stakeholders significantly influences both organizational identification and employees’ engagement, except for customers	Limited target population (Greater Cairo hotel workers), and focus on five-star hotels	Include expanding the target population, exploring other industries, and using diverse research methodologies​​
ElBelehy and Crispim^22^	Egypt	Identifies adopted social sustainability practices in Egypt and determines factors affecting their implementation, focusing on institutional and stakeholder theories	Mixed methods (qualitative and quantitative): the research collected 6 managers’ opinions to gain deeper insight through in-depth interviews and a questionnaire answered by a total of 187 practitioners (managers, supervisors, and employees) from hospitality and tourism supply chains in Egypt	Social sustainability practices are adopted but limited to legal requirements and brand policies. Local suppliers boost the adoption of social practices	The study’s primary focus on five-star hotels could potentially limit the generalizability of the findings. This narrow focus might not adequately represent the broader hospitality and tourism industry in Egypt	Expand the scope of research to investigate various sustainability dimensions
Zaki^16^	Egypt & Saudi Arabia	Assess circular economy practices in green hotels and the role of Industry 4.0 innovations on hotel performance. Develop a conceptual framework linking CE and HP	Quantitative approach: survey of 400 experienced hospitality professionals; data was analyzed using structural equation modeling	Circular economy practices significantly promote low-carbon behavior, and low-carbon behavior improves environmental performance. Finally, eco-friendly behavior strengthens the positive effect of low-carbon behavior on environmental performance	Focused on professionals’ views in two MENA countries; did not address consumer perspectives or long-term effects	Broaden to other regions, include consumer perspectives, and use longitudinal data

**Table 2:** Profile of interviewees and hotels involved.

Hotel	Interview			Hotel			
	Gender	Management area	Work experience	Location	Destination type	Category	Size
Phase 1: Pilot study							
Pilot 1	Female	Marketing and sales	Extensive	Aswan	Cultural	Local chain	Medium
Pilot 2	Male	Purchasing	Extensive	Aswan		International chain	Large
Phase 2: Main study							
ICHs.1	Male	Sustainable program	Extensive	Aswan	Cultural	International chain	Medium
	Male	Purchasing	Extensive				
ICHs.2	Male	Purchasing	Extensive	Cairo	Urban & Cultural	International chain	Large
	Female	Marketing and sales	Extensive				
ICHs.3	Male	Purchasing	Extensive	Cairo	Urban & Cultural	International chain	Large
	Female	Marketing and sales	Extensive				
LCHs5.1	Male	General	Extensive	Aswan	Cultural	Local chain 5-Star	Medium
	Female	Marketing and sales	Extensive				
LCHs5.2	Male	General	Extensive	Red Sea	Natural	Local chain 5-Star	Large
	Male	Purchasing	Extensive				
LCHs5.3	Male	General	Extensive	Luxor	Cultural	Local chain 5-Star	Medium
	Male	Purchasing	Extensive				
LCHs4.1	Male	Purchasing	Extensive	Red Sea	Natural	Local chain 4-Star	Large
	Male	Financial	Extensive				
LCHs4.2	Male	General	Extensive	Red Sea	Natural	Local chain 4-Star	Large
	Male	Purchasing	Extensive				
LCHs4.3	Male	General	Extensive	Luxor	Cultural	Local chain 4-Star	Medium
	Male	Purchasing	Extensive				

Notes: Work experience - Limited: < 2 years, Moderate: 2–5 years, and Extensive: > 5 years; Hotel size - small hotel up to 100 rooms, medium hotel: 100–300 rooms, large hotel: >300–500 rooms.

**Table 3:** Step-by-step process of thematic analysis Braun and Clarke^117^

Steps of thematic analysis	Description
Familiarizing oneself with the data	Significant time reading and re-reading the interview transcripts, taking notes on main ideas and thoughts, and keeping detailed records of all collected data
Developing initial codes	After data has been thoroughly examined and identify and develop the initial code
Searching for patterns or themes across all codes	Examine the initial codes to capture significant information related to the research question, such as SSCPs (significant statements or comments made by participants), drivers, and barriers. Comprehensive records of theme generation should be maintained
Reviewing and checking themes	Adjust and assess the initial themes, review them against the data, and ensure they accurately capture the essence of the research question
Defining and explaining themes	Once the themes have been reviewed and refined, defined, and explained them in detail; this may involve renaming themes, maintaining records of theme naming, and presenting distinct evidence to support each theme
Writing the description	Finally, explain and unfold the coding analysis process in detail, including explanations of the specifics of the research findings. It is important to provide clear and concise explanations in writing to ensure the research is accurately understood

#### 4-star local chain hotels

LCHs4 are more concerned with Social SPs related to employees’ wages (17 mentions), as the hotel management is committed to its employees and pays their salaries regularly, ensuring that the wages can meet the needs of the employees and their families. Further, the three LCHs4 are engaged in maintaining safety and health environment initiatives, including providing safety equipment, clean restrooms, and personal hygiene tools.

*“The hotel has a fair pay system*,* and the employees feel secure in their jobs. Providing a fair wage is important for ensuring that employees can support themselves and their families.”* (General Director, LCHs4*2).

Furthermore, equity practices, such as selecting the best candidates for the job, conducting fair assessments for all employees, promoting deserving employees, and ensuring equal treatment without discrimination, were emphasized. Ethics practices were also deemed essential, with all interviewees aware of a clear and transparent code of ethics. Employee welfare is prioritized through the provision of nutritious meals, equipped restaurants for employees, and access to health and social security benefits.

*“The hotel management is committed to following a code of ethics and ensuring fair and legal working conditions for employees.”* (General Director, LCHs4*2).

*“To satisfy the guests*,* we must satisfy the employees first*,* and we follow the principle of ‘we are gentlemen and gentlewomen*,* and we are serving gentlemen and gentlewomen.”* (Purchasing Director, LCHs4*3).

The prevention of forced labor was also emphasized: *“Before starting the work*,* the employees are informed of the salary they will receive*,* and the salary is appropriate for the tasks they will be performing.”* (Purchasing Director, LCHs4*1). Moreover, the LCHs4 demonstrate a commitment to preventing child labor and implement policies and practices that prohibit the employment of minors and ensure compliance with child labor laws.

Legal and regulatory requirements are also recognized, with hotels demonstrating a responsibility to comply with relevant laws and regulations, including those related to labor, safety, and environmental protection. On the other hand, women-centric initiatives received relatively little attention, as only two hotels (LCHs4*1, LCHs4*3) mentioned such initiatives. Interviewees stated that pregnant women are entitled to vacation, and breastfeeding women have their work schedules reduced to less than eight hours.

During interviews, managers of LCHs referred to the adoption of local sourcing practices, emphasizing the importance of supporting the local market, economy, and industry through local purchasing. Although prohibited practices of child and forced labor were mentioned 12 and 6 times, respectively, on-site inspections to verify compliance were not evident; the managers only stated that the hotel would cancel contracts with suppliers that breached this rule. Only one interviewee discussed health and safety practices for suppliers: *“Before choosing the suppliers we deal with*,* we make a field visit to the suppliers’ factories to ensure that the work environment is safe and that the principles of human rights are applied. For example*,* there are security and safety devices*,* and there are medical devices and first aid*, *as well as preliminary emergency exits.”* (Purchasing Director, LCHs4*3). As part of their community-level social sustainability programs, interviewees from 4-star local chain hotels mentioned philanthropy practices, such as establishing orphanages and helping charitable organizations, 7 times. Additionally, these hotels carry out activities such as providing relief to backward areas around the hotel and offering training and qualification programs for tourism students entering the workplace, in support of local schools and hospitals.

#### 5-star local chain hotels

The most prominent practices of Social SPs discussed by LCH5 managers are creating a safe and healthy work environment for employees (29 mentions), with a strong emphasis on training and discouraging harmful habits.

*“The hotel has also obtained the Industrial Safety Certificate*,* which means that the requirements for security and safety for the workers are available.”* (General Director LCHs5*1).

The three LCHs5 place a strong emphasis on ethical practices and regulatory responsibility. Interviewees emphasized the importance of adhering to the hotel’s code of ethics, which recognizes employees as partners in the workplace, as well as regulatory compliance practices, such as adherence to labor and wage laws.

*“Government agencies carry out periodic inspections of hotels to ensure that they are free of any labor violations.”* (General Director, LCHs5*2).

The hotels also prioritize the equitable treatment of employees and fair compensation based on their job responsibilities and seem to pay more attention to employee welfare than LCHs, as related issues are mentioned 11 times in the interviews. Basic training programs are provided to improve employee efficiency and performance:

*“One of the most important duties of the hotel towards employees is to create a training program to improve their level of performance”* (Purchasing Director, LCHs5*2).

The participants frequently mentioned the fair and equitable wages system implemented by the hotels, where salaries are distributed based on the amount of work performed by each employee. Women-centric initiatives are also evident in these hotels, including paid maternity leave. The hotels strictly adhere to the code of ethics and labor laws that prohibit child labor under the age of 18 and forced labor.

The LCHs5 demonstrated an inward-looking approach to implementing social practices, with a greater emphasis on practices within their organizations rather than in their supply chains. They shared similar concerns with the LCHs4 regarding local sourcing practices to encourage the national economy and the local community.

*“If you take a tour of the hotel*,* you will find that 98% of the products are local.”* (Marketing and Sales Director, LCHs5*1).

In terms of supplier audits and compliance verification, none of the hotel respondents reported any instances of auditing their supplier firms or canceling contracts with suppliers who breached regulations. Education and training for suppliers were also given relatively less emphasis; hence, they were described in only three passages by the representatives of LCHs 5*1 and LCHs 5*2.

At a community level, hotels donate and contribute to the construction of health units and hospitals, send food to orphanages, offer training to young people, and accept trainees from universities to prepare them for the workforce.

#### International chain hotels

Our study found that ICHs implement Social SPs carefully: these firms have implemented standardized procedures and documented HR policies and plans. Ethical practices were emphasized in 54 interview passages, reflecting the need for a strong moral code to motivate implementation.

*“We perform these practices as a professional and ethical commitment from the company*,* and this is by our code of ethics because we are one of the greatest hotel operators’ companies*,* not only in Egypt but worldwide.”* (Sustainable Program Director, ICH.1).

ICHs display a greater focus on safety and healthy environments, with the three hotels in the sample already certified with ISO 14,001 standards. The interviewed ICH managers also stated that employee welfare initiatives are prioritized, with a supportive and inclusive work environment that encourages growth, as mentioned in interviews 21 times. Additionally, these interviewees demonstrated a strong interest in the practice of fair wages, providing detailed answers and insights (mentioned 17 times) for equity practice. It is noteworthy that, overall, the case ICHs provide equal personal development opportunities and promote gender equality in the workplace. ICHs are more inclined towards the adoption and implementation of development and training. Women-centric initiatives compared to LCHs, as stated by the representatives of the hotels: *“The hotel ensures equal opportunity for males and females to further their careers*,* and we have a program to increase the employment of women.”* (Sustainable Program Director, ICH.1). The managers of ICHs emphasized their unwavering dedication towards implementing initiatives aimed at prohibiting child and forced labor.

As in the LCHs, ICHs highlighted the importance of compliance with laws and the Ministries’ regulations. ICHs demonstrate a more substantial commitment to promoting Social SPs within their hotels and throughout their supply chains and surrounding communities. One notable example of such initiatives is local sourcing, which not only helps to stimulate the local economy but also financially supports the local resources. The hotels ensure supplier health and safety, prevent child labor, and prohibit forced labor in supplier firms, providing education and training to suppliers.

*“As part of our 2025 Sustainability and Social Impact Goals*,* we aim to have 100% of our associates complete human rights training*,* including on human trafficking awareness*,* responsible sourcing*,* and recruitment policies and practices.”* (Purchasing Director, ICH.3).

The analysis of ICHs prioritizes philanthropy to support local communities. Education initiatives, such as internships and training, volunteering at local charities, and providing financial aid to schools, hospitals, orphanages, and homes for the elderly, are their key focus areas. They believe this investment strengthens relationships with stakeholders and contributes to their long-term sustainability.

*“There are several reasons why our hotel is motivated to participate in or create CSR programs and initiatives; firstly*,* we believe that it is our responsibility as a business to give back to the communities in which we operate.” (Marketing and Sales Director*,* ICH.3)*.

### Economic sustainability practices

**Fig. 5:**
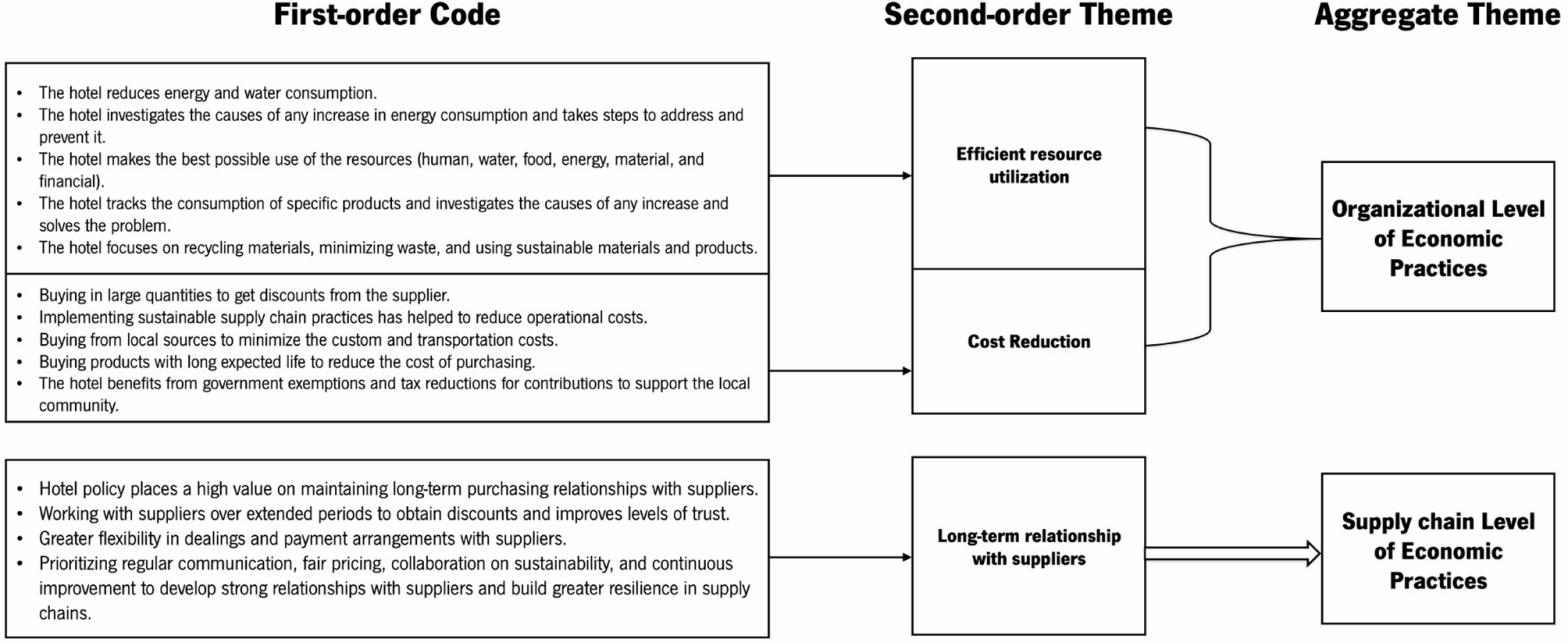
Coding structure of aggregate dimensions for economic pratices derived from interviews content.

Cost reduction, efficient resource utilization, and long-term relationships with suppliers are among the key economic sustainability practices that Egyptian hotels have adopted to achieve sustainable development goals (See Fig. 5). Our exploration of economic sustainability practices captures the coding structure of aggregate dimensions for economic practices by the hotels. Table ‎6 summarizes the number of mentions of economic sustainability practices by the managers of case hotels.

#### 4-star local chain hotels

LCHs4 managers emphasized cost reduction as an essential element of economic sustainability (8 mentions; 4 themes). The themes referred to include purchasing in bulk to obtain supplier discounts, buying products with a long lifespan to reduce operational costs, sourcing from local suppliers, and purchasing long-lasting products to reduce costs while protecting the environment.

*“It is preferable to buy products with a long-life cycle and buy them in large quantities to benefit from discounts from suppliers and to preserve the environment.”* (General Director, LCHs4*2).

LCHs4 prioritize energy conservation through various means, including the utilization of intelligent lighting systems and motion sensors, reducing energy and water consumption in bathrooms and kitchens, and segregating waste while promoting clean energy practices. Special devices and employee training ensure that water conservation and waste disposal are closely monitored to reduce costs. These methods were emphasized in interviews (mentioned 19 times), showing the importance of efficient resource utilization. Moreover, the study highlights the importance LCHs place on developing long-term supplier relationships (8 mentions), which provide numerous benefits, such as favorable treatment, extended payment periods offered by suppliers to hotels, and hotels providing crucial support to their suppliers during times of crisis. The hotels had been dealing with some suppliers for over two decades (including suppliers of fish, eggs, fruit, vegetables, meat, and poultry), demonstrating the positive outcomes of such relationships.

*“We have some suppliers that we have been working with for over twenty years*,* such as fish and egg suppliers. These long relationships allow us to get better treatment from our suppliers*,* and sometimes they allow us to have more time to pay our bills.”* (Purchasing Director, LCHs4*1).

#### 5-star local chain hotels

LCHs5 also prioritize cost reduction as an essential aspect of economic sustainability practices (8 mentions). Efficient resource utilization is crucial to hotel sustainability (37 mentions); these practices were categorized into waste management, energy and water conservation, and the use of biodegradable tools. The hotels have contracts with a water treatment plant for reusing sewage water and with a government-affiliated waste collection company for recycling. The hotels utilize water-saving tools and monitor resource consumption.

*“When the expected life of devices such as screens ends*,* we create a report*,* and the head of the department reviews it every 3 months. We sell them by the kilogram to electronics stores to be reused or recycled.”* (Purchasing director, LCHs5*2).

The LCH5 interviewees highlight the importance of building and maintaining long-term relationships with suppliers (11 mentions). These relationships rely on trust, shared interests, and flexibility, and offer benefits such as on-time delivery, discounts, and deferred payment. The hotel’s commitment to its suppliers is exemplified by its decision to pay part of its dues during the crisis. Some partnerships have endured for over 18 years, yielding preferential treatment, discounts, and mutual trust. Thus, hotels view their suppliers as partners and aim to cultivate long-term relationships for mutual benefit.

*“By working with suppliers over extended periods*,* we can reap numerous benefits*,* including obtaining discounts*,* postponing payment*,* and benefiting from a level of trust and mutual understanding that allows for greater flexibility in our dealings.”* (Purchasing Director, LCHs5*2).

#### International chain hotels

ICHs adopt similar cost-reduction practices as LCHs, such as buying in bulk and buying durable products. However, the interviewees highlighted an additional theme: the hotel’s benefits from government exemptions and tax reductions for contributions to support the local community. The interviews with ICHs also revealed a greater focus on cost reduction (16 mentions).

ICHs prioritize waste reduction and resource conservation as part of their cost-saving strategies (mentioned 46 times). These hotels utilize recycled products and paper packaging, and they have implemented waste management and pollution control systems. They reduce carbon intensity and plastic use, and evaluate the waste produced by restaurants and cafes for composting and biofuel production. The hotel tracks consumption and investigates any increase in usage to find a solution. By using sustainable materials, the hotel can achieve environmentally responsible operations while maximizing cost savings.

Based on interviews with ICH managers, it is evident that they prioritize maintaining long-term relationships with suppliers (mentioned 16 times). They offer benefits such as discounts, postponed payments, and support during crisis times. Sourcing locally helps to build stronger relationships, promoting transparency and accountability. To establish and strengthen these relationships, hotels must prioritize regular communication, fair pricing, sustainability collaboration, and continuous improvement. Ultimately, long-term purchasing relationships are essential for creating a sustainable and resilient supply chain.

*“By prioritizing regular communication*,* fair pricing*,* collaboration on sustainability*,* and continuous improvement*,* hotels can develop strong relationships with their suppliers and build greater flexibility in their supply chains.”* (Purchasing Director, ICH.3).

### Drivers of sustainable supply chain practices adoption

**Fig. 6:**
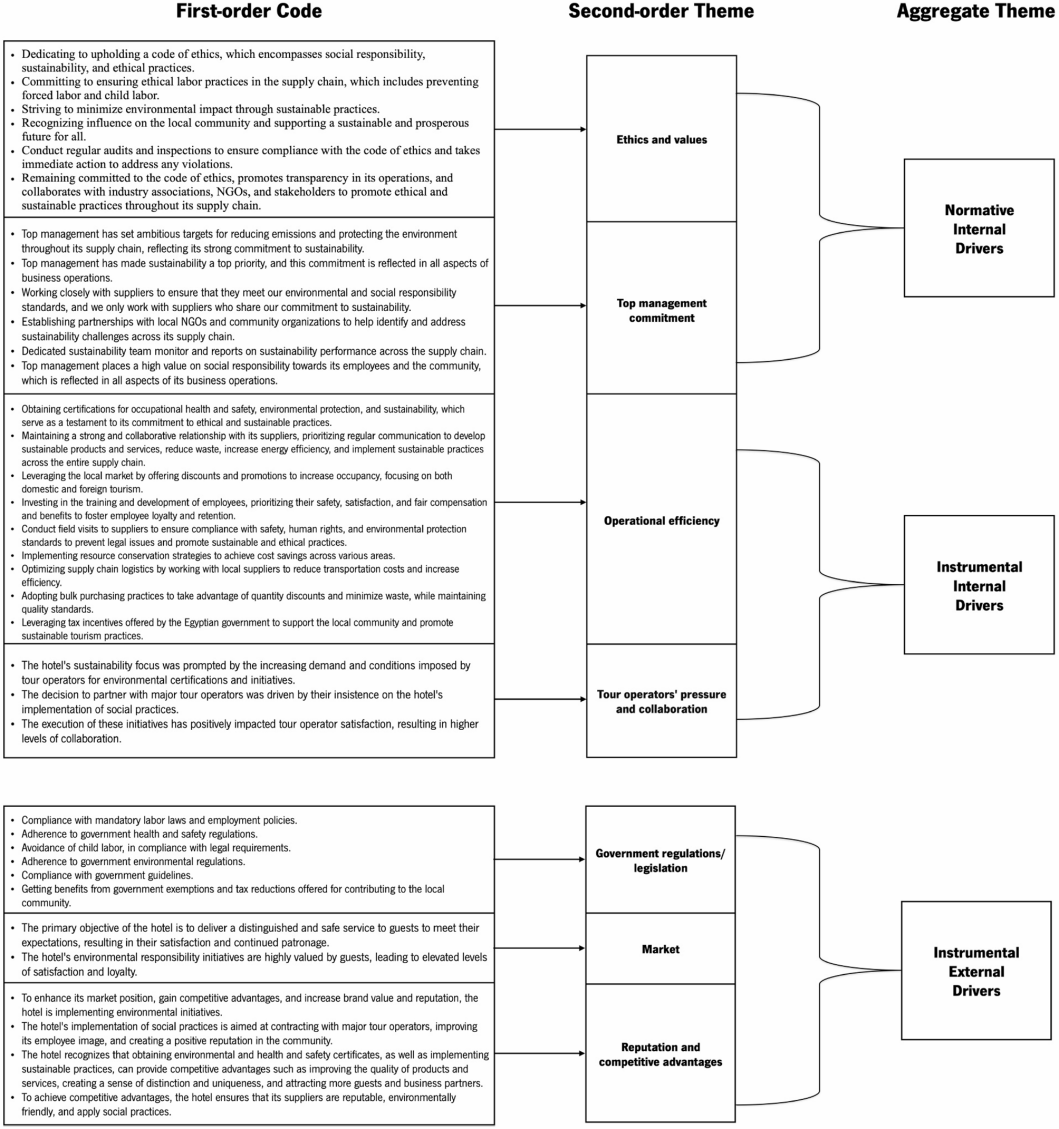
Coding structure of aggregate dimensions for drivers pratices derived from interviews content.

Our findings reveal that the drivers behind the adoption of SSCPs are diverse and can be classified into two main categories: internal and external drivers to the hotel supply chain. Internal drivers can be further subdivided into normative and instrumental drivers. Drivers related to supply chain partners were considered internal to highlight that, from a supply chain management perspective, it is the role of management to collaborate and expand on such drivers. A detailed list of the sustainable supply chain drivers under each dimension is provided in Fig. 6, and Table ‎7 summarizes the number of mentions of drivers by managers of case hotels. Specifically, the normative internal drivers of ethics and values, as well as top management commitment, and the instrumental internal drivers of operational efficiency, along with tour operators’ pressure and collaboration, were identified as motivating factors. External drivers, such as government regulations or legislation, market pressures, and reputation and competitive advantages, were also instrumental in driving SSCPs in these hotels.

#### 4-star local chain hotels

Representatives of LCHs4 stated internal ethics and values as crucial drivers for sustainable practices in the hospitality industry. They prioritize fair and legal working conditions for employees and refrain from dealing with suppliers employing children. The hotels’ code of ethics guides their social practices. These values were mentioned 15 times, which emphasizes their importance. Additionally, top management commitment was identified as a normative internal driver, as it was mentioned four times by managers of two hotels: LCHs4*1 and LCHs4*2.

In examining the instrumental internal drivers of sustainable practices in LCHs4, the most mentioned factor was operational efficiency (37 mentions). Due to limited resources, LCHs focus more on instrumental reasons, such as cost reduction, quality improvement, productivity increase, employee retention, sales growth, and attaining international certification. Hence, operational efficiency was the primary driver for the LCHs4 to implement sustainable practices. LCHs4 value their employees, collaborate with local suppliers, promote environmental awareness among staff, and reduce costs through bulk purchasing and long-term supplier relationships: *“We buy products with a long shelf life*,* as well as we prefer buying in large quantities to reduce the negative impact on the environment*,* order to face price fluctuations and to get a discount.”* (Purchasing Director, LCHs4*3). Tour operators’ pressure and collaboration are the second most instrumental internal drivers, with seven mentions. Collaboration with tour operators was a key motivator for the hotel to obtain environmental certifications and implement sustainable initiatives.

LCHs4 face significant challenges in implementing sustainable supply chain practices, with government regulations and legislation being the most frequently mentioned instrumental external driver (25 times). These hotels prioritize compliance with local environmental regulations to avoid penalties for non-compliance. Government agencies conduct regular audits to ensure compliance and prevent forced labor: *“There were also visits from the Ministry of Health and Tourism and the Ministry of Environment to ensure the safety of the hotel and its compliance with the standard health and environmental specifications set by the relevant ministries.”* (General Director, LCHs4*3). LCHs4 have adopted SSCPs due to other instrumental external drivers such as reputation and competitive advantages (20 mentions), as well as market pressure (11 mentions) driven by guests who expect sustainable initiatives and certifications.

#### 5-star local chain hotels

Ethics and values (20 mentions) are essential drivers for SSCPs in LCHs5. LCHs5t prioritize ethical labor practices and minimize their environmental footprint to uphold their code of ethics. Top management commitment was the least frequently mentioned driver (7 mentions), but an interviewee cited top management’s commitment as the primary reason for adopting sustainable practices.

Operational efficiency is the most important driver for SSCPs (52 mentions). Furthermore, the tour operators’ pressure and collaboration were frequently referred to (21 mentions). Partnership with major tour operators that insist on environmentally friendly practices has positively impacted guest and tour operator satisfaction, resulting in higher levels of loyalty.

*“The tour operator exerts great pressure on the hotel management to become environmentally friendly and sustainable*,* as most tour operators are from developed countries.”* (General Director, LCHs5*3).

Reputation and competitive advantages (32 mentions) are also instrumentally important external drivers of SSCPs for LCHs5:

*“The implementation of these initiatives benefits all elements of the supply chain. For example*,* the management of the hotel benefits from improving the overall level of cleanliness in the hotel and its ability to use these environmental initiatives as a marketing tool to improve the image and perception of the organization and thus increase the occupancy rate.”* (Marketing and Sales Director, LCHs5*1).

Government regulations and legislation (30 mentions) are significant instrumental external drivers of SSCPs for LCHs5. These hotels may be required to comply with local environmental regulations, and failure to do so may damage their reputation and result in penalties. Moreover they comply with mandatory labor laws and employment policies. In addition, the market (14 mentions) is considered an instrumental external driver. LCHs5 recognize that sustainable practices are becoming increasingly crucial to guest, who are more conscious of their impact on society and the environment.

#### International chain hotels

Ethics and values (59 mentions) are key drivers for SSCPs in ICHs: *“The main reason that motivates the hotel to apply these social responsibility initiatives is the moral commitment of the company and its code of ethics.”* (Sustainable Program Director, ICH.1). Top management commitment (48 mentions) is crucial to the success of sustainability programs, as it ensures the allocation of resources. The commitment is reflected in all aspects of business operations, including social responsibility towards employees and the community. All the ICH representatives stated that sustainability is a top priority for top management, and their commitment has been beneficial for their hotel.

*“It is clear that there is a strong moral commitment and sense of responsibility to spread environmental awareness in the community. This is a great motivation for our hotel to take steps to reduce our environmental impact.”* (Purchasing Director, ICH.3).

ICHs face unique challenges in implementing SSCPs due to strict corporate sustainability policies and targets. Operational efficiency is the most frequently mentioned driver (87 mentions). Furthermore, the tour operators’ pressure and collaboration (16 mentions) are considered a significant driver: *“Before the tour operator starts dealing with the hotel and signing contracts to provide us with guests*,* they send an auditor for inspection and review on the level of security and safety as well as environmental measures in the hotel.”* (Sustainable Program Director, ICH.1).

The market (11 mentions), is recognized as a key external driver for sustainability efforts: *“Many of our guests appreciate the efforts we make to be environmentally responsible*,* and this has resulted in higher levels of guest satisfaction and loyalty.”* (Marketing and Sales Director, ICH.3). Reputation and competitive advantages (24 mentions) were also referred to as drivers for sustainability. Although complying with mandatory regulations (20 mentions), these hotels prioritize self-regulation and internal inspections to implement sustainable practices: *“We place a strong emphasis on self-regulation and conduct comprehensive internal inspections to ensure adherence to sustainable practices.”* Marketing Director, ICH.2). Furthermore, the Ministries for Health, Environment, and Tourism have requirements and conduct regular inspections to check whether hotels are adhering to sustainable requirements.

### Barriers to sustainable supply chain practices adoption

**Fig. 7:**
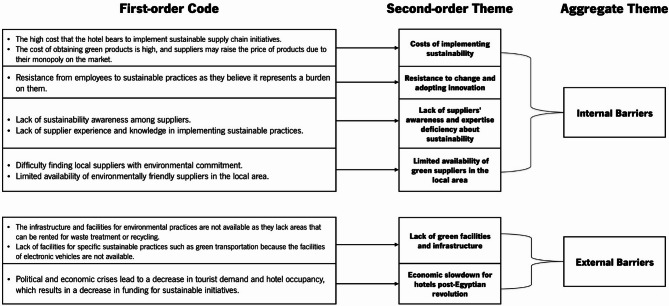
Coding structure of aggregate dimensions for barriers pratices derived from interviews content.

The research findings reveal that the barriers can be broadly categorized into two main groups: internal and external to the hotels’ supply chains. Obstacles related to supply chain partners were considered internal to highlight that, from a supply chain management perspective, issues related to those barriers should be managed. Internal barriers encompass topics such as the costs involved in implementing sustainability measures, resistance within the hotels to change and embrace innovations, and a deficiency in suppliers’ awareness and expertise regarding sustainability. Additionally, there is a challenge posed by the limited availability of local green suppliers. In contrast, the external barriers identified include the lack of green facilities and infrastructure in the wider area, including designated facilities for waste treatment or recycling, as well as the limited availability of electric vehicles, particularly in southern Egypt. Additionally, the environment and the economic downturn experienced by hotels following the Egyptian revolution. A detailed list of the identified SSC barriers is presented in Fig. 7, and Table 8 summarizes the number of mentions of various types of sustainability barriers by the managers of the case hotels.

**Table 4:** Mentions to environmental sustainability practices by managers of case hotels.

Environmental Sustainability	Hotel category		
	Local Chain Hotels 4* (n*m = 3*2 = 6)	Local Chain Hotels 5* (n*m = 3*2 = 6)	International Chain Hotels (n*m = 3*2 = 6)
Organizational level			
Waste Reduction Practices (c = 6)	10/36	23/36	32/36
Energy Use Reduction Practices (c = 2)	4/12	5/12	12/12
Water Use Reduction Practices (c = 3)	4/18	9/18	10/18
Reducing the Carbon Emission (c = 3)	0/18	0/18	12/18
Recycling and Reuse (c = 4)	2/24	8/24	12/24
Green Certifications (c = 2)	4/12	4/12	4/12
Green training (c = 2)	0/12	3/12	4/12
Supply chain level			
Green Supplier Selection (c = 7)	12/42	17/42	37/42
Green Purchasing (c = 7)	10/42	11/42	11/42
Green Supply Chain Collaboration with Suppliers (c = 3)	0/18	1/18	12/18
Green Supply Chain Collaboration with Tour Operators (c = 3)	9/18	11/18	13/18
Supply Chain Collaboration with Logistics and Green Providers (c = 5)	6/30	9/30	13/30
Green Supply Chain Collaboration with Government agencies (c = 3)	0/18	1/18	4/18
Collaboration with Green Consultant (c = 4)	2/24	6/24	1/24
Number of mentions by the interviewees	63/324	108/324	177/324

Notes: c - number of subcategories in the category; n - number of hotels from each category; m - number managers interviewed in each hotel; c*n*m = number of mentions if each manager mentions each subcategory once.

The number of mentions reflects the total frequency with which each practice was discussed by interviewees. Managers could refer to the same practice multiple times within a single interview, and each mention was counted separately. Thus, the totals may exceed the number of interviewees or hotels.

**Table 5:** Mentions to social sustainability practices by managers of case hotels.

Social sustainability	Hotel category		
	Local chain Hotels 4* (n*m = 3*2 = 6)	Local chain Hotels 5* (n*m = 3*2 = 6)	International chain Hotels (n*m = 3*2 = 6)
Organizational level			
Development and Training (c = 4)	6/24	11/24	12/24
Equity (c = 6)	15/36	12/36	16/36
Ethics (c = 2)	12/12	20/12	54/12
Employees Welfare (c = 3)	10/18	11/18	21/18
Maintaining Health and Safety Environment (c = 4)	16/24	29/24	29/24
Wages (c = 4)	17/24	12/24	17/24
Prohibition of Child labor (c = 2)	5/12	6/12	9/12
Prohibition of Forced labor (c = 2)	10/12	8/12	7/12
Regulatory Responsibility (c = 2)	11/12	14/12	9/12
Women Centric Initiatives (c = 6)	4/36	5/36	11/36
Supply chain Level			
Local sourcing (c = 2)	12/12	12/12	22/12
Education and Training for Suppliers (c = 2)	0/12	3/12	8/12
Ensuring Health and Safety (c = 2)	1/12	0/12	13/12
Prohibition of Child labor (c = 2)	12/12	5/12	21/12
Prohibition of Forced labor (c = 2)	6/12	3/12	16/12
Community Level			
Philanthropy (c = 7)	12/42	17/42	32/42
Number of mentions by the interviewees	149/312	168/312	297/312

Notes: c - number of subcategories in the category; n - number of hotels from each category; m - number managers interviewed in each hotel; c*n*m = number of mentions if each manager mentions each subcategory once.

The number of mentions reflects the total frequency with which each practice was discussed by interviewees. Managers could refer to the same practice multiple times within a single interview, and each mention was counted separately. Thus, the totals may exceed the number of interviewees or hotels.

**Table 6:** Mentions to economic sustainability practices by managers of case hotels.

Economic sustainability	Hotel category		
	Local Chain Hotels 4* (n*m = 3*2 = 6)	Local Chain Hotels 5* (n*m = 3*2 = 6)	International Chain Hotels (n*m = 3*2 = 6)
Organizational level			
Cost reduction (c = 5)	8/30	8/30	16/30
Efficient resource utilization (c = 5)	19/30	37/30	46/30
Supply chain level			
Long-term relationship with suppliers (c = 4)	8/24	11/24	16/24
Number of mentions by the interviewees	35/84	56/84	78/84

Notes: c - number of subcategories in the category; n - number of hotels from each category; m - number managers interviewed in each hotel; c*n*m = number of mentions if each manager mentions each subcategory once.

The number of mentions reflects the total frequency with which each practice was discussed by interviewees. Managers could refer to the same practice multiple times within a single interview, and each mention was counted separately. Thus, the totals may exceed the number of interviewees or hotels.

**Table 7:** Mentions to sustainability drivers by managers of case hotels.

Drivers	Hotel category		
	Local Chain Hotels 4* (n*m = 3*2 = 6)	Local Chain Hotels 5* (n*m = 3*2 = 6)	International Chain Hotels (n*m = 3*2 = 6)
Normative internal drivers			
Ethics and values (c = 6)	15/36	20/36	59/36
Top management commitment (c = 6)	4/36	7/36	48/36
Instrumental internal drivers			
Operational efficiency (c = 9)	37/54	52/54	87/54
Tour operators’ pressure and collaboration (c = 3)	7/18	21/18	16/18
Instrumental external drivers			
Government regulations/ legislation (c = 6)	25/36	30/36	20/36
Market (c = 2)	11/12	14/12	11/12
Reputation and competitive advantages (c = 4)	20/24	32/24	24/24
Number of mentions by the interviewees	118/216	176/216	265/216

Notes: c - number of subcategories in the category; n - number of hotels from each category; m - number managers interviewed in each hotel; c*n*m = number of mentions if each manager mentions each subcategory once.

The number of mentions reflects the total frequency with which each practice was discussed by interviewees. Managers could refer to the same practice multiple times within a single interview, and each mention was counted separately. Thus, the totals may exceed the number of interviewees or hotels.

**Table 8:** Mentions of sustainability barriers by managers of case hotels.

Barriers	Hotel category		
	Local Chain Hotels 4* (n*m = 3*2 = 6)	Local Chain Hotels 5* (n*m = 3*2 = 6)	International Chain Hotels (n*m = 3*2 = 6)
Internal barriers			
Costs of implementing sustainability (c = 2)	5/12	6/12	2/12
Resistance to change and adopting innovation (c = 1)	6/6	2/6	1/6
Lack of suppliers’ awareness and expertise deficiency about sustainability (c = 2)	0/12	2/12	5/12
Limited availability of green suppliers in the local area (c = 2)	2/12	1/12	3/12
External barriers			
Lack of green facilities and infrastructure (c = 2)	0/12	0/12	7/12
Economic slowdown for hotel post-Egyptian revolution (c = 1)	4/6	1/6	0/6
Number of mentions by the interviewees	17/60	12/60	18/60

Notes: c - number of subcategories in the category; n - number of hotels from each category; m - number managers interviewed in each hotel; c*n*m = number of mentions if each manager mentions each subcategory once.

The number of mentions reflects the total frequency with which each practice was discussed by interviewees. Managers could refer to the same practice multiple times within a single interview, and each mention was counted separately. Thus, the totals may exceed the number of interviewees or hotels.

#### 4-star local chain hotels

The most frequently stated challenges by the managers of the three LCHs were resistance to change and the adoption of innovation (6 mentions), which was attributed to a lack of awareness among hotel employees about the benefits of sustainable practices, with some perceiving them as a burden. The cost of implementing sustainability (5 mentions) was also identified as a significant hindrance. Interviewees stated that the high cost of purchasing new equipment and green products (e.g., green cleaning products, energy-efficient lighting, etc.) prevented the implementation of sustainable practices.

*“Resistance from hotel employees may also stem from concerns about the additional workload or changes in their job responsibilities that may be required.”* (General Director, LCHs4*3).

*“There may be some potential downsides*,* such as initial investment cost; hence*,* depending on the specific practices being implemented*,* there may be upfront costs associated with purchasing new equipment or green products*,* or implementing new processes. While these costs may be offset in the long term by cost savings or other benefits*,* they can still be a barrier to implementation.”* (General Director, LCHs4*2).

Furthermore, managers from two hotels (the Purchasing Director of LCHs4*2 and the General Director of LCHs4*3) stated that one of the internal barriers they faced while implementing sustainable practices was the limited availability of green suppliers in the local area, especially at the beginning of their green initiatives.

*“Now there are no difficulties*,* but at the beginning*,* there was a difficulty in finding suppliers who were committed to environmental preservation and offered environmentally friendly products.”* (Purchasing Director, LCHs4*2).

In the context of external barriers, the economic slowdown for hotels post-Egyptian revolution affected the funding process for environmental and social initiatives: *“These costly initiatives have been interrupted on occasion due to a decrease in tourist demand caused by political and economic crises.”* (General Director, LCHs4*3).

#### 5-star local chain hotels

In LCH5, the most mentioned barrier to SSCPs is the high cost of implementation, mentioned 6 times. Representatives from three LCHs5 reported that some sustainable initiatives are costly, and after the Egyptian crisis period, the hotels focused on paying off old debts. Some board members viewed these initiatives as too expensive.

*“In 2019*,* when the hotel’s occupancy rate returned*,* we were busy paying off old debts*,* so we were not interested in environmental initiatives due to the high cost. In my opinion*,* we have lost the term ‘environmentally friendly hotel’*,* which was one of our most important marketing tools. As a result*,* the hotel has lost a significant competitive advantage.”* (General Director, LCHs5*1).

Additionally, resistance to change from the employees also led to an unfavorable environment for sustainability, where this barrier was mentioned twice: *“The culture of the employees was against the implementation of the practices*,* as we made a great effort to train them on the implementation of these initiatives*,* and continuous control was required. For example*,* regarding waste segregation in the hotel*,* we placed cameras to ensure that the employees followed the proper procedures for waste segregation.”* (General Director, LCHs5*1).

Furthermore, the lack of awareness and expertise among suppliers regarding sustainability was mentioned by two interviewees (the General Director of LCHs5*1 and the Purchasing Director of LCHs5*3). Additionally, the Marketing and Sales Director of LCHs5*1 emphasized the limited availability of green suppliers in the local area.

Among the external barriers, the economic slowdown post-Egyptian revolution is significant hurdle to implementing sustainable practices in Egypt: *“However*,* due to the Egyptian revolution in 2011 and the resulting increase in the cost of these sustainable initiatives*,* we were unable to complete the sustainable plan as tourism in Egypt stopped.”* (Marketing and Sales Director, LCHs5*1).

#### International chain hotels

In interviews with ICH managers, internal barriers to achieving sustainability goals were identified as the costs of sustainability and employee resistance to change. However, when compared to local hotels, sustainability costs were only mentioned twice and employee resistance to change was only mentioned once by the same hotel (ICH. 3). This suggests that ICHs may have stronger capabilities and more resources to overcome these challenges, possibly due to their ability to select highly qualified employees and invest significantly in their training and development.

One of the main internal barriers faced by ICHs is a Lack of suppliers’ awareness and expertise deficiency about sustainability, as mentioned 5 times. Interviewees from the three hotels reported low supplier awareness: “*The low awareness of suppliers can be a major obstacle to implementing sustainable practices in the supply chain.”* (Purchasing Director, ICH.3). Furthermore, the limited availability of green suppliers in the local area affected the implementation of environmental practices in hotels as mentioned by the Purchasing Directors of ICH.1 and ICH.2.

The lack of green facilities is considered a significant external barrier, mentioned 7 times by ICH’s managers. The lack of waste treatment facilities, green infrastructure, and recycling areas was identified as a challenge for sustainable practices. Additionally, the absence of green transportation for suppliers such as electric vehicles: *“Similarly*,* the absence of green infrastructure*,* such as renewable energy sources or sustainable waste management systems*,* can make it challenging for hotels to adopt sustainable practices and reduce their environmental impact.”* (Marketing Director, ICH.2).

## Discussion and conceptual framework

### Discussion

This work corroborates existing studies by Xu and Gursoy^119^, Babu et al.^120^, Modica et al.^81^, and Pereira et al.^100^. However, this study differs from these studies in three ways. First, we present sustainable supply chain practices (SSCPs) in terms of their three dimensions of sustainability and their impact on hotels’ environmental, social, economic, operational, and reputational performances. Second, we identify the commonalities and differences between international chain hotels (ICHs), 5-star local chain hotels (LCHs5), and 4-star local chain hotels (LCHs4). Third, we focus on the SSCPs at multiple levels. While sustainable practices in hotels from developed countries have been extensively studied, there is an increasing recognition of the importance of investigating sustainable practices in hotels from developing countries, which have unique economic, social, and environmental challenges that require tailored solutions. Although our study is not the first to highlight the perspectives of hotels in a developing country, to the best of our knowledge, it is the first study to examine the differences and similarities between ICHs, LCHs5, and LCHs4 in developing economies, focusing on all three dimensions of sustainability. This approach gains relevance in light of the findings by Devesa and Peñalver^121^, who argued that higher-category hotels, such as ICHs and LCHs, tend to be more technically efficient due to the intense pressure they face to maintain their competitive position and star ratings. This efficiency imperative likely influences their adoption and implementation of sustainable practices, an aspect our study explores across different hotel categories. Although Robin et al.^19^ attempted to explore the distinct approaches of mature and emerging destinations, they focused on comparing mature destinations from a developed country (Spain) and emerging destinations from a developed country (Chile).

#### Environmental sustainability practices

The integration of sustainability principles into the supply chain management of the hospitality industry has become increasingly important in recent years^122^. While there is a significant amount of research that has been conducted regarding green practices in the hotel industry, e.g., Al-Aomar and Hussain^123^, and social sustainability, e.g., Alameeri et al.^28^, there has been a dearth of studies that focus on the three dimensions of sustainability comprehensively^28^. Acampora et al.^124^ conducted a systematic literature review that analyzed 600 papers published in the field of sustainability within the hospitality industry. This review found that 66.17% of the papers had an environmental approach to sustainability, with 25 papers combining this approach with economic considerations, and 13 papers combining it with social considerations. The lack of a holistic approach hinders the effectiveness of sustainability efforts, as neglecting one dimension may result in unintended consequences on the others. For example, while reducing water consumption is an effective environmental sustainability strategy, it may lead to guest dissatisfaction if it negatively impacts their experience. Therefore, hotels must consider all three dimensions of sustainability in the hotel supply chain to ensure a comprehensive and balanced approach towards sustainability. Therefore, we considered SSCPs in terms of their three dimensions of sustainability.

In line with previous literature Cerchione and Bansal^20^, Peña-Miranda et al.^29^, Chen and Chen^53^, Modica et al.^81^, Merli et al.^119^, and Peña-Miranda et al.^126^, we found various dimensions of SHSCPs (see Figs. 3, 4, and 5). Our results reveal that both the number of SSCPs and the extent of their formalization differ across ICHs, LCHs5, and LCHs4. The implementation of organizational environmental practices by hotels is an essential component in promoting environmental sustainability. However, the priorities for environmental practices vary across the three types of hotels studied. Our study found that ICHs and LCHs share convergence in environmental practices, including waste reduction, reduced water use, recycling and reuse, and green training. This presence indicates that these practices are becoming more prevalent in the hotel industry, regardless of hotel ownership or star rating. As travelers become more environmentally conscious, hotels may be motivated to adopt sustainability practices to attract and retain customers^47^. Moreover, the growing global concern over climate change and the need to reduce environmental impacts may influence many hotels to prioritize environmental sustainability. However, the study also found notable differences in the performance of LCHs4 in these initiatives. The observed lag suggests that greater investment and commitment to sustainability practices may be necessary for this group of hotels.

Regarding energy use reduction practices, international chain hotels (ICHs) reported full adoption with 12/12 mentions, compared to local chain hotels (LCHs 4* and 5*) with only 4/12 and 5/12 mentions, respectively (Table 4). This suggests resource and budget constraints limit energy-saving efforts in local hotels. For green certifications, all hotel categories showed similar adoption levels (4/12 mentions each), indicating certification is now a standard industry practice influenced by tour operator pressures (see Table 7). Finally, carbon emission reduction practices appeared only in ICHs (12/18 mentions), highlighting that larger hotels with more resources implement advanced environmental initiatives, unlike smaller local chains (Tables 4 and 8).

In terms of green supplier selection at the supply chain level, international chain hotels (ICHs) demonstrated notably higher performance, with 37 mentions out of 42, compared to local chain hotels (LCHs) 4* at 12 mentions and LCHs 5* at 17 mentions (Table 4). This difference likely reflects the ICHs’ more substantial commitment to top management sustainability and the greater availability of resources for implementing sustainable supply chain practices (SSCPs). Regarding green supply chain collaboration with government agencies, ICHs again performed significantly better (4/18 mentions) than LCHs (4* and 5*, 0/18 and 1/18, respectively), indicating more proactive engagement with regulatory and policy actors, which supports advanced SSCP implementation. Similarly, for collaboration with suppliers, ICHs showed superior performance (12/18 mentions) versus LCHs 4* (0/18) and 5* (1/18), reflecting ICHs’ larger supplier networks and greater bargaining power, enabling more effective sustainable collaboration. In contrast, collaboration with logistics and green providers revealed convergence between ICHs (13/30) and LCHs (5/30), while LCHs lagged (6/30), suggesting that such collaborations might be more accessible or prioritized across hotel types. However, smaller local 4-star chains still face challenges.

In green supply chain collaboration, the practices of tour operators and green purchasing converged in performance across all hotel categories. This suggests that these practices are becoming more widespread across the industry, regardless of hotel ownership or star rating. Finally, in supply chain collaboration with green consultant practices, a clear difference was observed between LCH5 and both ICHs and LCHs4, with the former performing significantly better. This may reflect the high level of competition among LCHs, as these hotels often compete with ICHs, and they may feel greater pressure to improve their sustainability performance. However, when examining collaboration with green consultants, the data reveal a distinct pattern: 5-star local chain hotels (LCHs5) reported engaging with green consultants more frequently (6 mentions) compared to international chain hotels (ICHs, 1 mention) and 4-star local chain hotels (LCHs4, 2 mentions), as shown in Table 4. This suggests that, while the use of green consultants is not widespread across all hotel types, LCHs5 are more proactive in seeking external expertise to enhance their sustainability efforts. The relatively limited engagement with green consultants among ICHs may be attributed to their established internal sustainability policies and greater in-house resources, allowing them to advance environmental practices without extensive reliance on external consultants. For LCHs4, fewer mentions may reflect resource constraints, which can limit their ability to invest in external consultancy services. Thus, although green consultants are recognized in sustainability literature as valuable partners, the actual data from this study indicate their involvement is currently modest and varies notably by hotel category.

Xu and Gursoy^119^ revealed that energy use reduction, water use reduction, waste reduction, carbon emission reduction, as well as recycling and reuse, are organizational environmental practices in the USA. Similarly Modica et al.^81^; Hussain et al.^11^ identified organizational environmental practices in the UAE and Italy. Additionally, at the supply chain level of environmental practices, Modica et al.^81^, Abdou et al.^82^, Xu and Gursoy^119^, and Hussain et al.^11^ found that purchasing green products was the only sustainable practice employed by the hotel. Our work sheds light on additional supply chain levels of environmental practices, such as green collaboration with suppliers, green collaboration with tour operators, collaboration with consultants on green practices, collaboration with logistics and green providers, and green collaboration with government agencies, which are specific to our study of Egyptian hotels.

Our investigation into the practices of environmental sustainability in the Egyptian hotel industry aligns with and expands recent scholarly findings. Abdou et al.^82^ reported a trend toward the increased adoption of eco-conscious practices among Egypt’s top-rated, eco-friendly five-star hotels, including the use of energy-conserving appliances, fixtures that reduce water usage, and effective waste management systems. These actions, echoing our research findings, signal a growing commitment to environmental ethics in the Egyptian hospitality sector. Furthermore, Abdou et al.^82^ examined the implementation of green practices in four and five-star Egyptian hotels, finding a pronounced commitment to environmental sustainability. The authors’ study revealed notable disparities in the extent of green practices based on star rating, with hotels rated higher demonstrating more extensive environmental efforts. This commitment to environmental sustainability is central to the implementation of these practices, underscoring a profound dedication to ecological responsibility. Such findings provide essential corroboration and additional depth to our research, broadening our understanding of the diverse sustainable practices employed by hotels of different calibers in Egypt.

#### Social sustainability practices

International chain hotels (ICHs) outperform both 5-star and 4-star local chains (LCHs5 and LCHs4) in key organizational social practices, including ethical conduct (54/12 mentions), employee welfare (21/18), and women-centric initiatives (11/36), according to coded interview frequencies (Table 5). This pattern suggests that ICHs have established more comprehensive policies and resources in these areas. However, LCHs5 surpass LCHs4 in ensuring workplace health and safety (29/24 mentions vs. 16/24) and in supporting employee development and training (11/24 vs. 6/24) (Table 5). This likely reflects the higher budget allocation by LCHs5 for training and development, as reported by interviewees, enabling more effective staff empowerment and skill enrichment.

The data reveal that international chain hotels (ICHs), 5-star local chains (LCHs5), and 4-star local chains (LCHs4) exhibit similar performance regarding core fair labor practices, including employee equity (ICHs: 16/36, LCHs5: 12/36, LCHs4: 15/36), prohibition of child labor (ICHs: 9/12, LCHs5: 6/12, LCHs4: 5/12), prohibition of forced labor (ICHs: 7/12, LCHs5: 8/12, LCHs4: 10/12), and regulatory responsibility (ICHs: 9/12, LCHs5: 14/12, LCHs4: 11/12) (Table 5). This convergence arises because local and international labor laws and regulations often require these practices. As a result, hotels, regardless of their rating or ownership, must comply with these laws and regulations to avoid legal consequences and maintain their reputation. Additionally, there is an increasing demand from customers and stakeholders for ethical and socially responsible business practices, which leads hotels to prioritize these practices in order to maintain their reputation and brand image. Nevertheless, the differences in the extent to which hotels enforce and implement these practices could be attributed to the resources and size of the hotels, as well as the level of commitment and investment in sustainability and social responsibility by their top management.

Interview data reveal significant differences in supply chain social practices among international chain hotels (ICHs), 5-star local chains (LCHs5), and 4-star local chains (LCHs4). ICHs consistently outperformed both local chain categories on key measures—such as local sourcing (ICHs: 22/12 mentions vs. LCHs5: 12/12 vs. LCHs4: 12/12), ensuring suppliers’ health and safety (ICHs: 13/12, LCHs5: 0/12, LCHs4: 1/12), and education and training for suppliers (ICHs: 8/12, LCHs5: 3/12, LCHs4: 0/12). ICHs also had the highest compliance for prohibiting child and forced labor practices at their suppliers (child labor – ICHs: 21/12, LCHs5: 5/12, LCHs4: 12/12; forced labor – ICHs: 16/12, LCHs5: 3/12, LCHs4: 6/12). Interestingly, LCHs4 outperformed LCHs5 in areas such as prohibiting suppliers’ use of child and forced labor, even with fewer resources. This suggests that LCHs4 may prioritize focused interventions—such as enforcing labor standards—where their impact is most pronounced, rather than spreading limited resources across a broader range of practices. Conversely, LCHs5 did not emphasize ensuring suppliers’ health and safety, while LCHs4 were less involved in providing education and training for suppliers’ sustainability. These performance patterns may stem from differences in resource allocation and strategic focus. While ICHs’ superior results reflect their robust resources and established supply chain policies, LCHs appear to target practices—such as upholding labor standards—where their engagement can be most effective, given financial constraints. This focused approach may allow LCHs4 to excel in specific supply chain social practices, despite its limitations. However, resource differences and managerial priorities across hotel types remain only one potential explanation for these observed variations.

International chain hotels (ICHs) exhibit the highest engagement in community-level social practices, with 32 out of 42 coded mentions of philanthropic activities, followed by 5-star local chains (LCHs5) with 17 out of 42 and 4-star local chains (LCHs4) with 12 out of 42 (Table 5). This performance gap likely reflects ICHs’ greater financial capacity and broader networks, which enable more extensive and coordinated philanthropic initiatives. LCHs5 also prioritize philanthropy, viewing it as a strategic approach to uphold their luxury reputation and foster customer loyalty. In contrast, LCHs4 engage less in these practices, consistent with their smaller size and limited resources. These findings are grounded in the frequency of mentions across interview data, illustrating how resource availability and strategic priorities shape social community engagement among hotel categories.

Peña-Miranda et al.^29,49^ have delineated several organizational-level social practices within the context of Colombia, while Farmaki et al.^126^ have identified similar social practices within the context of Greece. These practices encompass employee development and training, employee welfare, equity, maintaining a safe and healthy work environment, fair wages, and respect for human rights. Alameeri et al.^28^ identified similar organizational-level practices in the UAE, including ethics practices. At the supply chain level, the importance of local sourcing and supplier training was highlighted. For the community level, Peña-Miranda et al.^29^, Ghaderi et al.^34^, Gürlek and Tuna^48^, Chandran and Bhattacharya^58^, Alameeri et al.^28^, Peña-Miranda et al.^126^, and Inoue and Lee^129^ identified several philanthropy practices for the surrounding community.

The findings of our study shed light on additional social practices implemented by Egyptian hotels at the organizational level. These practices include regulatory responsibilities and initiatives that prioritize women (e.g., paid leave for pregnant women and reduced work schedules for breastfeeding women to less than eight hours), which are described for the first time in the context of Egyptian hotels. Furthermore, at the supply chain level, Egyptian hotels have demonstrated efforts to ensure health and safety conditions for suppliers, as well as initiatives aimed at prohibiting child and forced labor within their supply chains. These practices highlight the specific approaches adopted by Egyptian hotels to address social responsibility and ethical concerns within their organization and supply chain operations.

The current study elucidates a pronounced integration of social sustainability practices in Egypt’s hotel industry, with a particular emphasis on ethical standards, employee well-being, and women-centric initiatives. Contrarily, the existing corpus of literature presents a dichotomous view. ElBelehy and Crispim^22^ reveal, in their examination of five-star hotels in Egypt, an erratic and non-systematic implementation of social sustainability protocols, with notable deficiencies within the Hospitality and Tourism Supply Chain. The respondents’ demographics in this study comprised over 55% of employees and supervisors; this composition may offer insight into the disparity observed in comparison to our results, as their viewpoints and experiential knowledge markedly differ from those in higher echelons of management. Additionally, ElBelehy and Crispim^22^ utilized social media as a platform to disseminate the questionnaire for data collection. This method introduces inherent biases, as respondents’ self-selection may not be representative of the entire spectrum of stakeholders within the industry and may raise concerns regarding the reliability and validity of the data collected.

Furthermore, the absence of differentiation among hotel categories in their questionnaire meant that responses could encompass a broad spectrum of hotels from various star ratings, without a clear distinction based on their classification. ElBelehy and Crispim^22^ highlight significant lacunae in areas such as health and safety, ethical conduct, anti-corruption, and human rights, advocating for heightened governmental intervention to augment efficacy. Conversely, the findings of Ibrahim et al.^23^ align with our research, signaling an upward trend in Corporate Social Responsibility (CSR) practices in the Egyptian hotel industry. The analysis by these authors represents an avant-garde approach to social sustainability, aligning with our findings on the progressive social undertakings in this sector. This spectrum of scholarly opinions accentuates the complexity and varied implementation of social sustainability efforts in Egypt’s hospitality industry, thus necessitating further investigation.

#### Economic sustainability practices

Interview data show that both international chain hotels (ICHs) and local chain hotels (LCHs) are converging in their adoption of key economic sustainability practices. This includes widespread use of efficient resource utilization and organizational cost reduction (ICHs: 46/30 and 16/30 mentions; LCHs5: 37/30 and 8/30; LCHs4: 19/30 and 8/30), as well as a shared emphasis on developing long-term supplier relationships at the supply chain level (ICHs: 16/24, LCHs5: 11/24, LCHs4: 8/24) (Table 6). These patterns indicate that sustainable management of resources and partnerships with suppliers are becoming industry standards, driven by growing awareness of their strategic importance for competitiveness and resilience. However, notable differences persist, especially in the economic performance of LCHs (4-star local chains), which lag behind their international and 5-star local counterparts in terms of resource efficiency and supplier relationships. The superior performance of ICHs in these areas is likely attributable to their larger resource base and greater economies of scale, enabling more substantial investment in cost-saving and supplier management initiatives. This supports the interpretation that while economic sustainability practices are spreading across Egypt’s hotel sector, organizational capacity and scale continue to drive performance gaps among hotel categories.

Cerchione and Bansal^20^ and Xu and Gursoy^28^ and have also identified resource utilization practices and cost reduction as organizational economic sustainability practices in the USA and India. Chen and Chen^53^ outline the same economic sustainability practices, focusing on the relationship with stakeholder practices in Taiwan. Our work has different results in that cost reduction, relationship with suppliers such as buying in large quantities to get discounts from the supplier, buying products with long expected life to reduce the cost of purchasing, achieving benefits from government exemptions and tax reductions for contributions to support the local community, and flexibility in dealings and payment arrangements with suppliers are highlighted.

#### Drivers of sustainable supply chain practices adoption

This study contributes to filling a gap in sustainability research by linking its drivers to stakeholder theory, as it is expected that Egyptian LCHs and ICHs implement SSCPs due to stakeholder pressure^128^. We found that the perception of stakeholder pressures and their effectiveness varied among ICHs, LCHs, and LCHs, as presented in Table 9. This variation in perception shows the complexity of stakeholder influences in different hotel categories. Our findings indicate that these hotels adopt SSCPs not only for instrumental reasons, such as strategic benefits and competitive advantage, but also for normative reasons, driven by ethical considerations and corporate values. This approach highlights the importance of considering both strategic and ethical dimensions in adopting sustainable practices in the hospitality sector. This result supports the fundamental concept of normative stakeholder theory, which prioritizes the interests of all stakeholders regardless of the outcomes^129^.

**Table 9:** Perceived stakeholders’ pressure for sustainable supply chain practices.

Stakeholders	4-star Hotel	5-star Hotel	International Hotels
Managers	Low	Low	High
Employees	Low	Low	Low
Guests	Moderate	High	Moderate
Tour Operators	Moderate	High	Moderate
Suppliers	Low	Low	Low
Government	High	High	Moderate
Competitors	Moderate	High	Moderate
Community	Low	Low	Low

Note: Involvement categories (“low,” “moderate,” “high”) are based on the relative frequency of stakeholder mentions in interviews, corresponding roughly to < 33%, 33–67%, and > 67% of coded references.

Our results determined that the normative drivers in the Egyptian hotel sector are ethics and values, which is consistent with Asadi et al.^21^, Chandran and Bhattacharya^58^, and Morales-Contreras et al.^61^, and top management commitment, which is consistent with Khatter et al.^33,62^ and Shah^130^. Our findings also strengthen the existing instrumental logic that has been described in the literature that a business’s primary purpose is to attain performance targets, such as operational efficiency, reputation, and competitive advantages, and government regulations/legislation^21,33,58,61,62,130,131^. Furthermore, we contributed to the existing literature by identifying pressures from various stakeholder groups and highlighting the differences in ICHs, LCHs5, and LCHs4.

Table 9 highlights significant differences in stakeholder influence within the hotel supply chain, particularly contrasting the roles of suppliers and tour operators. The categorization of stakeholder involvement into “low,” “moderate,” and “high” is based on the frequency of coded mentions by interviewees, with thresholds defined as follows:

- Low: stakeholders mentioned in less than 33% of relevant codes,
- Moderate: mentioned in 33–67% of codes,
- High: mentioned in over 67% of codes.

According to this classification, suppliers have a relatively limited influence across all hotel categories (ICHs, LCHs 5, LCHs 4), as reflected by consistently low involvement scores. In contrast, tour operators demonstrate strong influence, particularly on 5-star local chains (LCHs5), where their involvement is categorized as “high” due to frequent references as key drivers of sustainable supply chain practices. This disparity highlights the crucial role of tour operators in shaping sustainability initiatives, which is likely driven by their position as intermediaries between hotels and international markets, as well as their capacity to enforce sustainability standards. This clear differentiation of stakeholder impact provides actionable insight into where hotels and policymakers may strategically focus efforts to enhance sustainable supply chain adoption, emphasizing the leverage held by tour operators over suppliers.

Our findings indicate that different drivers hold varying significance for each hotel type, as reflected in the coded data. Operational efficiency is the most critical internal driver for international chain hotels (ICHs), with 87 mentions out of a possible 54, compared to 52/54 for 5-star local chains (LCHs5) and 37/54 for 4-star local chains (LCHs4). This underscores that ICHs prioritize operational efficiency to support strict corporate sustainability targets, cost reduction, and increased profitability. Tour operators’ pressure and collaboration are most influential for LCHs5 (21/18 mentions), followed by ICHs (16/18), and least for LCHs4 (7/18). This trend suggests that higher-rated local hotels are exceptionally responsive to tour operators, integrating their expectations into sustainable practices. Ethics and values are more prominent for ICHs (59/36 mentions), with lower counts for LCHs5 (20/36) and LCHs4 (15/36), reflecting the need for ICHs to maintain a global reputation for ethical and socially responsible operations. Lastly, top management commitment emerges as a major driver in ICHs (48/36), compared to just 7/36 for LCHs5 and 4/36 for LCHs4. This highlights the centrality of leadership and strategic decision-making in advancing sustainability in ICHs, while this factor is less emphasized in local chains.

Our analysis reveals that among external drivers, 5-star local hotels (LCHs5) place the strongest emphasis on market forces, reputation, and competitive advantage as motivators for adopting sustainable supply chain practices (SSCPs), with 14/12 mentions for market and 32/24 for reputation and competitive advantages—distinctly higher than both international chain hotels (ICHs: 11/12 for market, 24/24 for reputation/competitive advantages) and 4-star local chains (LCHs4: 11/12 for market, 20/24 for reputation/competitive advantages) (Table 7). This suggests that LCHs5 particularly leverage sustainability for strategic positioning and branding. Additionally, government regulations and legislation are more critical for LCHs, with 25/36 mentions for LCHs4 and 30/36 for LCHs5, compared to 20/36 for ICHs (Table 7). Two main factors likely explain this difference: (1) ICHs commonly rely on self-regulation and internal auditing, drawing on established international sustainability frameworks and corporate strategies, thus experiencing less external pressure; (2) LCHs face stricter monitoring by government agencies, which conduct regular audits to ensure compliance—mainly to prevent forced labor—making regulatory adherence a pronounced driver for these hotels.

#### Barriers to sustainable supply chain practices adoption

The most evident internal barrier in the Egyptian hotel sector is the cost of implementation, which is consistent with Khatter et al.^33^, Chan et al.^65^, Luo et al.^66^, Kasim^131^, and Chan^132^, and the other barrier is resistance to change and adopting innovation which corroborates existing studies by Khatter et al.^33^, Chan et al.^65^. Our research findings indicate that the absence of green facilities (e.g., facilities designated for waste treatment or recycling) and the limited availability of electric^131^ serve as external barriers to the implementation of SSCPs.

We identified several additional barriers unique to the hotel industry within the Egyptian context: the lack of knowledge and awareness about sustainability among suppliers as an internal barrier, and the economic slowdown for the hotel following the Egyptian revolution as an external barrier. Collaborative efforts with supply chain partners, such as joint sustainability training and shared resource utilization initiatives, can play a pivotal role in mitigating these barriers, promoting a more comprehensive adoption of SSCPs across the supply chain.

Insufficient knowledge and awareness of sustainability among suppliers create a significant obstacle to acquiring sustainable products, thereby diminishing the overall sustainability of the supply chain. When suppliers lack a comprehensive understanding of sustainability principles or their significance, hotels face difficulties in obtaining eco-friendly goods and services, which undermines their environmental efforts. To address this issue, it is crucial to implement targeted training programs for suppliers, foster collaboration between hotels and their supply chain partners, and establish clear sustainability criteria for selecting and evaluating suppliers. These actions not only enhance supplier capabilities but also ensure that supplier practices align with the sustainability objectives of the hotel industry, thereby improving the resilience and performance of the supply chain. Insufficient knowledge and awareness of sustainability among suppliers create a significant obstacle to acquiring sustainable products, thereby diminishing the overall sustainability of the supply chain. When suppliers lack a comprehensive understanding of sustainability principles or their significance, hotels face difficulties in obtaining eco-friendly goods and services, which undermines their environmental efforts. To address this issue, it is crucial to implement targeted training programs for suppliers, foster collaboration between hotels and their supply chain partners, and establish clear sustainability criteria for selecting and evaluating suppliers. These actions not only enhance supplier capabilities but also ensure that supplier practices align with the sustainability objectives of the hotel industry, thereby improving the resilience and performance of the supply chain^133^.

The economic downturn that followed the Egyptian revolution had a significant impact on the hotel industry, resulting in decreased revenues, stalled investments in sustainability efforts, and extensive job losses. The drop in international tourism and heightened financial uncertainty compelled numerous hotels to reduce expenses and delay or abandon environmental projects. To counter these challenges, industry stakeholders and policymakers need to implement financial incentives—such as tax reductions or grants—specifically designed to support sustainability during economic downturns. Furthermore, hotels should establish robust crisis management strategies and explore revenue diversification options, such as expanding non-room services, to maintain financial stability and ensure ongoing investment in sustainable practices during challenging economic times^134^.

The barrier of costs associated with implementing sustainability is notably more pronounced for 4-star and 5-star local chains (LCHs4 and LCHs5), with 5/12 and 6/12 coded mentions, respectively, compared to only 2/12 mentions for international chain hotels (ICHs) (Table 8). This disparity likely reflects the more limited financial resources available to local chains, which face greater difficulty investing in sustainable supply chain practices (SSCPs) that require substantial upfront costs. Similarly, employee resistance to change and innovation adoption emerges as a more significant barrier for LCHs4, supported by 6 out of 6 mentions, compared to 2 out of 6 for LCHs5 and 1 out of 6 for ICHs. The less sophisticated management structures and limited experience with sustainability initiatives in LCHs4 may contribute to heightened resistance among employees when adapting to new sustainable practices. These results suggest that resource constraints and organizational maturity significantly impact the ability of local hotels to overcome barriers to the implementation of SSCP. In contrast, ICHs’ greater financial and managerial capabilities mitigate these challenges.

Barriers such as the lack of green facilities and infrastructure (7/12 coded mentions for ICHs, compared to 0/12 for both LCHs5 and LCHs4), suppliers’ limited awareness and expertise in sustainability (5/12 mentions for ICHs, 2/12 for LCHs5, and 0/12 for LCHs4), and the limited availability of green suppliers (3/12 for ICHs, 1/12 for LCHs5, 2/12 for LCHs4) have a more significant impact on international chain hotels (ICHs) than on local chains (Table 8). This is primarily because ICHs often operate on a larger scale across multiple regions, encountering wide variability in infrastructure quality and stakeholder readiness. The diverse operational footprint of ICHs makes achieving consistent SSCP implementation more complex, as they must coordinate with suppliers who possess different levels of sustainability knowledge and capacity. As a result, successful adoption of sustainable supply chain practices in ICHs depends not only on their resources but also on heightened collaboration, supplier education, and overcoming infrastructure deficits in the markets where they operate.

### Conceptual framework

**Fig. 8:**
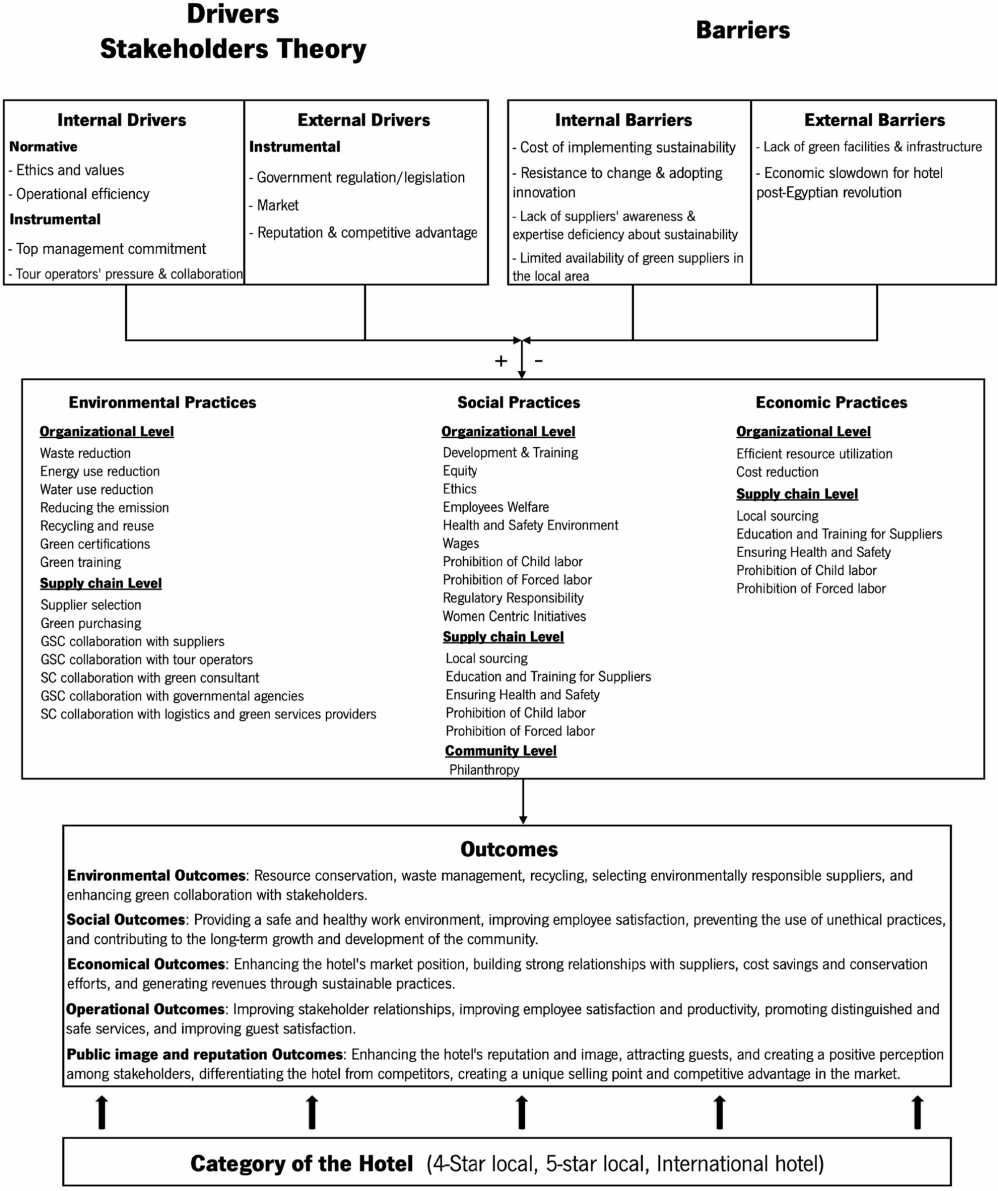
Conceptual framework.

Based on our findings and informed by stakeholder theory, we developed a conceptual framework (Fig. 8) to present the sustainability phenomenon at local chain hotels (4-star and 5-star) and ICHs in an integrated manner. This framework, developed in the Egyptian context, may help explain SHSCM in developing countries by incorporating drivers, barriers, SSCPs, and outcomes for ICHs and LCHs. Key aspects of this framework include:

- **Integration of Internal and External Factors**: We categorized the drivers of SSCPs into internal and external to the supply chain, drawing on both instrumental and normative stakeholder theories. This categorization offers a comprehensive view of the motivators behind SSCP implementation, highlighting the balance between ethical obligations and business objectives.
- **Identification of Barriers**: The framework distinguishes between internal and external supply chain barriers, providing insights into the specific challenges faced by hotels in adopting sustainable practices, thereby contributing to a deeper understanding of the hurdles to sustainability implementation in the hotel supply chain.
- **Outcomes of SSCPs**: The framework encompasses various outcomes of SSCPs, categorized as environmental, social, economic, operational, and reputation based. This holistic approach aligns with the triple bottom line concept and extends the understanding of sustainability impacts in the hotel supply chain.
- **Comparison Across Hotel Categories**: A novel aspect of our framework is the comparison of drivers, barriers, SSCPs, and outcomes across different hotel categories (ICHs, LCHs5, and LCHs4), providing a nuanced view of sustainability in diverse hotel contexts.

The framework (Fig. 8) not only synthesizes our findings but also contributes to the literature by offering a structured approach to understanding sustainability in the hotel supply chain. It highlights the importance of considering a broad range of stakeholders and provides a basis for comparative analysis across different hotel types. By focusing on both the internal and external dimensions of sustainability drivers and barriers, the framework adds depth to the existing understanding of sustainability in the hotel supply chain, particularly in developing countries like Egypt.

#### How the conceptual framework was built

The conceptual framework presented in Fig. 8 is developed following a rigorous theory-building approach commonly adopted in qualitative research literature^135^. Instead of simply deriving themes from interview coding, we triangulated our primary interview data with existing key theories and empirical studies in sustainable hospitality supply chain management (e.g., Zaki^16^, Asadi et al.^21^, ElBelehy and Crispim^22^, Freeman^26^, Khatter et al.^33,62^, Elkington^73^. This allowed us to construct a holistic model that positions sustainable supply chain practices (SSCPs) as the core phenomenon, influenced by clearly defined antecedents (drivers and barriers) and resulting in multidimensional consequences (environmental, social, economic outcomes), consistent with recent calls for integrating stakeholder theory and the triple bottom line in hospitality research^62,136^.

To provide theoretical clarity, the final framework (Fig. 8) moves beyond the first-order and second-order coding and instead organizes the emergent concepts into three main components:

- Antecedents: These are the drivers and barriers to SSCP adoption, grounded both in the data and in prior frameworks (e.g., institutional theory, stakeholder theory, and resource-based views); in particular, our research is explicitly grounded in stakeholder theory. Antecedents are grouped into internal (ethics and values, top management commitment, operational efficiency, tour operators’ pressure and collaboration) and external (regulatory pressure, market competition, reputation, and competitive advantages) factors, as established in past studies Asadi et al.^21^, Khatter et al.^33^.
- *Core Phenomenon*: The central element of the framework is the implementation of Sustainable Supply Chain Practices (SSCPs) in hotels, as informed by aggregate environmental, social, and economic practices found both in extant literature, such as, Cerchione and Bansal^20^, Peña-Miranda et al.^29^, and Farmaki et al.^126^, and our empirical data.
- *Consequences*: These encompass the multidimensional outcomes of SSCP adoption for hotels, covering environmental (resource conservation, waste management, recycling etc.), social (providing a safe and healthy environment, improving employee satisfaction etc.), economic (cost savings, competitive advantage etc.), operational (improving stakeholder relationships, improving productivity, and improving guest satisfaction etc.), and Public image and reputation (enhancing reputation and image, attracting guests, and creating a positive perception etc.). This outcome-oriented perspective is emphasized in the literature as crucial for theory testing and policy guidance^16,20,23^.

#### Application of the framework in a real-life setting

In the next section, the Framework is applied in a hotel. The Sustainable Supply Chain Practices (SSCPs) framework for hotels is a management-oriented, practical guideline that enables hotel managers to identify the drivers of sustainability with practical impact systematically, the practices that must be implemented, and the expected challenges and benefits. Here is how it can be applied in practice:

*Diagnosing Drivers and Barriers (Antecedents)*.

- **Practical Implications**: Management can employ the Framework by determining which drivers are most salient to their setting and by prioritizing sustainability initiatives accordingly. For example, if regulatory compliance is a significant motivator, the hotel might focus on achieving (or even surpassing) the required standards as the starting point for further action.

*Adopting Core Sustainability Processes (Core Phenomena)*.

- **Environment Practices**: This gives hotels a way to benchmark their current initiatives, such as waste minimization, energy efficiency, and eco-purchasing.
- **Social Practices**: Hotels are also advised to support employee well-being and local procurement and to participate in community activities.
- **Business Practice**: Hotels can leverage the Framework to concentrate on reducing costs and developing long-term relations with suppliers.

*Understanding and Leveraging Consequences*.

- **Enhanced Reputation**: Hotels with strong sustainability communication, such as promoting green certifications or community philanthropy, experience added value from competitive advantage and better relationships with their stakeholders.
- **Cost Saving**: Effective resource usage and wastage minimization all resulted in reduced working costs, something evident in both international and local chains.
- **Iterative Process**: The model stresses the consideration of barriers (e.g., cost, employee resistance) and the need to modify strategies over time.

*Hotel Management and Implications for the Sustainability Strategies*.

- **Strategic Synchronization**: This means managers can synchronize their sustainability actions both to outside pressure (other regulations and market forces) and to internal ability, which implies that the firm is both in compliance and that it is also adding value.
- **Resource Allocation**: Hoteliers can allocate resources more effectively by knowing which practices matter most (waste reduction, community engagement, etc.).
- **Benchmarking and Goal setting**: The tool offers a structure for benchmarking existing sustainability practices against industry standards and for setting trackable sustainability goals.
- **Stakeholder Engagement**: It is believed that the focus on community-level procedures should help domestic hotels to develop better relationships with local stakeholders, which would help in improving their social license to operate.

## Conclusion

This study explored sustainable supply chain practices (SSCPs) among Egyptian hotels, focusing on international chain hotels (ICHs), 5-star local chain hotels (LCHs5), and 4-star local chain hotels (LCHs4). Drawing on qualitative data from nine case studies, the research provides nuanced insights into the adoption, drivers, and barriers of SSCPs in a developing country context.

The findings indicate that while all hotel categories have adopted some form of sustainable supply chain practices, the scope and sophistication of these initiatives vary. ICHs generally demonstrate more advanced and formalized practices, particularly in areas such as waste management, energy efficiency, and supplier engagement. LCHs5 tend to focus on cost-saving measures and basic recycling, whereas LCHs4 prioritize straightforward waste reduction and local sourcing, often constrained by limited resources. These variations reflect differences in resource availability, stakeholder pressures, and organizational priorities rather than any inherent superiority of one hotel type over another.

The study also highlights the diverse drivers motivating sustainability adoption. Both normative factors (such as ethics and values) and instrumental considerations (including operational efficiency and stakeholder pressures) play a role, with their relative importance differing across hotel categories. Notably, tour operators and regulatory requirements emerge as significant external influences, especially for local chains. However, the extent to which these drivers translate into comprehensive sustainability initiatives is shaped by each hotel’s context and capacity.

Barriers to SSCP implementation are evident across all hotel types but manifest differently. Financial constraints, resistance to change, and knowledge gaps are particularly pronounced among local chains, while ICHs more frequently cite challenges related to supplier awareness and infrastructure limitations. These obstacles underscore the importance of context-specific strategies and the need for targeted support, particularly for resource-constrained hotels.

While the study suggests potential associations between SSCP adoption and improvements in environmental, social, economic, operational, and reputational outcomes, it is essential to recognize the exploratory nature of the research. The qualitative, multi-case approach offers in-depth, context-rich insights but does not support broad generalizations or claims of universal effectiveness. The results are best interpreted as indicative of patterns and relationships within the sampled hotels, rather than as definitive evidence of causality or superiority.

In summary, the research contributes to the understanding of sustainable supply chain management in the hospitality sector by illuminating both commonalities and differences among hotel categories in Egypt. The integration of stakeholder theory offers a valuable lens for understanding the interplay between ethical imperatives and performance goals. However, further research—including studies in other contexts, hotel categories, and with broader stakeholder perspectives—is needed to deepen and validate these findings. Collaboration among hotels, supply chain partners, and policymakers remains essential for advancing sustainability in the sector, particularly in developing economies where contextual challenges are significant.

## Limitations and future research

### Limitations

This study has several limitations. The qualitative, multiple-case study approach with a small sample of nine hotels in Egypt restricts the generalizability of the findings. The results may not be representative of the broader hotel sector in Egypt, nor are they directly applicable to other regions or hotel types. The selection of hotels based on accessibility and willingness to participate may have introduced a selection bias, potentially favoring hotels that are more engaged in sustainability. Reliance on qualitative interviews and self-reported data introduces response and social desirability biases, and the absence of data triangulation (such as direct observation or document analysis) may affect the robustness of the findings. Additionally, the Egypt-specific context means that cultural, economic, and regulatory factors unique to this setting may further limit the applicability of the results elsewhere.

### Future research recommendations

To address the research limitations, future research should expand the sample size and diversity by including a wider range of hotel types, ownership structures, and geographic locations. Cross-country comparative studies are especially recommended to assess the influence of different cultural and regulatory environments and to strengthen the generalizability of findings. Employing quantitative or mixed-methods approaches would help validate and expand upon these qualitative results. Incorporating additional data sources—such as direct observation, document analysis, or third-party assessments—can strengthen the credibility of future findings. Research should also include hotels less engaged in sustainability to capture a broader range of challenges and motivations. Future studies are encouraged to propose and investigate specific research questions such as: “How does the adoption of eco-labels influence customer trust and booking decisions in the hotel sector?”, “What are the best practices and barriers to implementing sustainable certifications across different hotel categories?”, and “How can circular economy models be operationalized within hotel supply chains to minimize waste and maximize resource use?”. Finally, future studies should explore the role of technology and digital transformation in sustainable supply chain practices, as digital tools and innovation are increasingly recognized as key drivers of sustainability in the hospitality sector.

## Implications

### Theoretical implications

This study contributes to the literature on sustainable supply chain practices (SSCPs) in the hospitality sector, particularly within developing country contexts. By applying stakeholder theory, the research highlights how varying stakeholder pressures and organizational capacities influence the adoption and implementation of SSCPs across different hotel categories. The findings demonstrate that sustainability drivers and barriers are not uniform, but are shaped by hotel type, ownership structure, and local context. The conceptual framework developed in this study provides a foundation for future research to investigate further the interplay between stakeholder expectations, organizational resources, and sustainability outcomes in the hospitality and related service industries. Additionally, the study highlights the importance of considering contextual factors—such as economic instability and regulatory environments—when examining SSCPs in emerging markets.

### Practical implications

The research provides actionable recommendations for hotel managers and industry stakeholders, especially in developing countries:

- Staff Training and Awareness: Implement regular training programs to enhance employee knowledge and engagement with sustainable practices, focusing on areas such as waste management, energy efficiency, and water conservation.
- Supplier Collaboration: Foster strong relationships with suppliers by setting precise sustainability requirements, offering joint training, and prioritizing partnerships with local and committed suppliers.
- Certifications and Partnerships: Pursue green certifications and collaborate with tour operators, government agencies, and NGOs to boost credibility and attract environmentally conscious guests.
- Resource-Efficient Technologies: Invest in energy-saving and waste-reduction technologies, starting with cost-effective measures like LED lighting or recycling programs, and scale up as resources allow.
- Overcoming Barriers: Seek out government incentives, grants, or industry partnerships to address financial constraints, and participate in knowledge-sharing forums to bridge information gaps.
- Leadership and Culture: Integrate sustainability into the hotel’s core values, with visible support from top management, and recognize staff contributions to encourage widespread adoption.
- Monitoring and Communication: Track key sustainability metrics and communicate progress transparently to both staff and guests, building trust and enhancing reputation.

## Supplementary Information

Below is the link to the electronic supplementary material.

Supplementary Material 1

## Data Availability

“The data that support the findings of this study are available from the corresponding author upon reasonable request. Researchers interested in accessing the data may contact us for further details.”
