# Supplementary material for: Sustainable supply chain practices of local versus international hotels: multiple case studies in Egypt
Source: Sci Rep. 2025 Sep 15;15:32542. doi: 10.1038/s41598-025-18819-9 (PMC12436614; doi:10.1038/s41598-025-18819-9)
Supplement: Supplementary file 1 — Supplementary Material 1 [file 41598_2025_18819_MOESM1_ESM.docx]

# **Appendix A - Semi-Structured Interview Protocol**

**Introduction**

This study aim is to investigate the adoption of Sustainable Supply Chain Management (SSCM) practices amongst hospitality organizations in Egypt and its impact on firms' sustainable performance. By sustainable, I mean focusing on meeting the needs of the present without compromising the ability of future generations to meet their needs. The concept of sustainability is composed of three pillars: economic, environmental, and social.

**The objectives of this part of the study are: -**

- 1. To identify the current sustainability practices on supply chains of the Egyptian hospitality industry;
  2. To identify enablers and the barriers to applying sustainable practices in hospitality SC; and
  3. To gather information to, in the next phase of the study, apply a questionnaire-based survey to test and evaluate an integrated model to study the influence of SHSCM practices on the organizations’ sustainable performance.

**General guidelines: -**

- Please remember that there are no right and wrong answers, and the aim of the interview is to understand your experiences and opinions.
- My estimation is that the interview will take approximately 60 minutes.
- I need to record the interview to be able to transcribe your opinions and responses for data analysis purposes. The recording is just for research purposes, will only be accessed by me and it will be safeguarded, and I will send you a script of the interview before I start analyzing it to get your feedback.

**Please note the following: -**

- The interview will be confidential and no names (e.g., yours or of the company) will be made public.
- Names will be changed when the research is transcribed thus total anonymity can be ensured.
- The results of the research will present data in an aggregated way.
- The interview is voluntary, and you may end the interview at any time.
- You also have the right to decline to answer any specific questions and you can raise questions or concerns whenever necessary.
- Three years after the thesis defense, all interview recordings will be destroyed.

Do you approve that this interview is recorded?

If the participant does not agree that the interview is recorded:

I understand that you do not feel comfortable with me recording this interview so I will need to take notes. These notes will be used for data analysis and data coding purposes. The notes are just for research purposes, will only be accessed by me will be safeguarded.

May you please sign the informed consent form so we can continue with the interview.

**General Information**

Organizations’ name:

Size of the organization (Turnover):

Department:

Respondent position in the organization:

Age of the respondent:

Gender of the respondent:

Qualification / work experience of the respondent:

**Questions**

1) Can you please describe your function and one of your typical workdays?

2) What are your organization’s procedures concerning the environment in: -

- supplier selection;
- products’ choice in purchasing;
- reverse logistics & waste management;
- collaboration with third parties providing services.

3) Can you describe any specific initiatives your organization has implemented to reduce energy, water, and food consumption?

4) How do you monitor and measure your organization's energy, water, and food usage regularly?

5) Is there any green collaboration between your organization and suppliers to improve environmental practices and standards? (If the answer is affirmative,) please describe specific examples of these collaborations. (If the answer is affirmative,) Why?

6) Does your organization collaborate with tour operators to promote responsible and eco-friendly practices? (If the answer is affirmative,) please describe specific examples of these collaborations. (If the answer is affirmative,) Why?

7) How does your organization work with government agencies to comply with environmental regulations and support local sustainability efforts?

8) Why is each type of practice used? And how long has the company applied for each of them?

9) Can you describe what type of difficulties has the company encountered in applying each of these environmental practices?

10) What have been the advantages of the application of each of these environmental practices?

11) What have been the disadvantages of the application of each of these environmental practices?

12) Who or what has influenced the organization to implement the environmental practices referred to?

13) Has your organization obtained any green certifications? If yes, which ones and what were the driving factors behind seeking these certifications?

14) What specific challenges has the company faced in implementing environmental practices in its supply chain?

15) How does your organization invest in the development and welfare of its employees?

16) Are there any specific programs or initiatives in place to promote equity, ensure compliance with labor laws, and support women in the workplace? (If the answer is affirmative,) please describe specific examples of these initiatives. (If the answer is affirmative,) Why?

17) Have you known about the use of child labor in your supply chain (e.g., by your suppliers)? And by your competitors?

18) And what about the use of forced labor in your supply chain (e.g., by your suppliers)? And by your competitors?

19) Does your organization have formal procedures concerning: -

- avoiding child labor in the supply chain (What is the minimum work age in your organization? And in the supply chain?);
- avoiding forced labor in the supply chain;
- human rights;
- assuring fair wages.

20) Does your organization provide education and training to its suppliers to ensure they meet the hotel's standards? (If the answer is affirmative,) please describe specific examples of these initiatives. (If the answer is affirmative,) Why?

21) How does your organization collaborate with suppliers to maintain health and safety standards throughout the supply chain?

22) Why did the hotel implement those social practices?

23) Does your organization favor local sourcing? (If the answer is affirmative,) for what types of purchases? (If the answer is affirmative,) Why?

24) Does your organization have other practices to support the local Egyptian communities? Can you describe them?

25) Who or what has influenced the organization to implement the social practices referred to?

26) What motivated the organization to participate in/ create those programs/ initiatives?

27) Can you describe what type of difficulties has the company encountered in applying each of these social practices?

28) Can you outline the main drawbacks or impediments encountered by the company in the application of social initiatives throughout its supply chain?

29) What strategies does your organization employ to achieve cost reduction without compromising the quality of services provided to guests?

30) Could you provide specific examples of cost-saving initiatives that have been implemented successfully within your organization?

31) Do your organizations have practices concerning maintaining long-term purchasing relationships? (Please describe each one of them)
